# Retinal photographs to predict life’s essential 8 for cardiovascular risk stratification: a novel deep-learning-based tool

**DOI:** 10.1093/ehjdh/ztag041

**Published:** 2026-03-06

**Authors:** Chenkai Zhao, Zheng Yong, Weiyuan Zhu, Suhang Mao, Hengheng Wu, Huimin Zhou, Ce Xu, Qihang Cang, Yongfu Yu, Yan Zong, Zijin Wang, Hu Liu, Jiong Li, Jiangbo Du

**Affiliations:** Department of Epidemiology, Center for Global Health, School of Public Health, Nanjing Medical University, 101 Longmian Avenue, Nanjing, Jiangsu 211166, China; Taizhou People's Hospital, Affiliated to Nanjing Medical University, 366 Taihu Road, Taizhou, Jiangsu 225300, China; Stomatological College, Nanjing Medical University, No.1 Shanghai Road, Nanjing, Jiangsu 211166, China; State Key Laboratory of Reproductive Medicine and Offspring Health, Nanjing Medical University, 101 Longmian Avenue, Nanjing, Jiangsu 211166, China; Department of Epidemiology, Center for Global Health, School of Public Health, Nanjing Medical University, 101 Longmian Avenue, Nanjing, Jiangsu 211166, China; State Key Laboratory of Reproductive Medicine and Offspring Health, Nanjing Medical University, 101 Longmian Avenue, Nanjing, Jiangsu 211166, China; Stomatological College, Nanjing Medical University, No.1 Shanghai Road, Nanjing, Jiangsu 211166, China; State Key Laboratory of Reproductive Medicine and Offspring Health, Nanjing Medical University, 101 Longmian Avenue, Nanjing, Jiangsu 211166, China; Department of Epidemiology, Center for Global Health, School of Public Health, Nanjing Medical University, 101 Longmian Avenue, Nanjing, Jiangsu 211166, China; State Key Laboratory of Reproductive Medicine and Offspring Health, Nanjing Medical University, 101 Longmian Avenue, Nanjing, Jiangsu 211166, China; Department of Epidemiology, Center for Global Health, School of Public Health, Nanjing Medical University, 101 Longmian Avenue, Nanjing, Jiangsu 211166, China; State Key Laboratory of Reproductive Medicine and Offspring Health, Nanjing Medical University, 101 Longmian Avenue, Nanjing, Jiangsu 211166, China; Department of Epidemiology, Center for Global Health, School of Public Health, Nanjing Medical University, 101 Longmian Avenue, Nanjing, Jiangsu 211166, China; State Key Laboratory of Reproductive Medicine and Offspring Health, Nanjing Medical University, 101 Longmian Avenue, Nanjing, Jiangsu 211166, China; Stomatological College, Nanjing Medical University, No.1 Shanghai Road, Nanjing, Jiangsu 211166, China; Stomatological College, Nanjing Medical University, No.1 Shanghai Road, Nanjing, Jiangsu 211166, China; Department of Biostatistics, School of Public Health, Fudan University, 220 Handan Road, Shanghai 200032, China; Department of Epidemiology, Center for Global Health, School of Public Health, Nanjing Medical University, 101 Longmian Avenue, Nanjing, Jiangsu 211166, China; State Key Laboratory of Reproductive Medicine and Offspring Health, Nanjing Medical University, 101 Longmian Avenue, Nanjing, Jiangsu 211166, China; Department of Ophthalmology, Suzhou Municipal Hospital, the Affiliated Suzhou Hospital of Nanjing Medical University, 242 Guangji Road, Suzhou, Jiangsu 215002, China; Department of Ophthalmology, the First Affiliated Hospital with Nanjing Medical University, 300 Guangzhou Road, Nanjing, Jiangsu 210029, China; Department of Ophthalmology, the First Affiliated Hospital with Nanjing Medical University, 300 Guangzhou Road, Nanjing, Jiangsu 210029, China; Department of Epidemiology, Center for Global Health, School of Public Health, Nanjing Medical University, 101 Longmian Avenue, Nanjing, Jiangsu 211166, China; State Key Laboratory of Reproductive Medicine and Offspring Health, Nanjing Medical University, 101 Longmian Avenue, Nanjing, Jiangsu 211166, China; Department of Epidemiology, Center for Global Health, School of Public Health, Nanjing Medical University, 101 Longmian Avenue, Nanjing, Jiangsu 211166, China; Taizhou People's Hospital, Affiliated to Nanjing Medical University, 366 Taihu Road, Taizhou, Jiangsu 225300, China; State Key Laboratory of Reproductive Medicine and Offspring Health, Nanjing Medical University, 101 Longmian Avenue, Nanjing, Jiangsu 211166, China; Henan Branch of State Key Laboratory of Reproductive Medicine and Offspring Health, the Third Affiliated Hospital of Zhengzhou University, No.7 Frontkangfu Street, Zhengzhou, Henan 450052, China

**Keywords:** Deep learning, Retina, Life’s Essential 8, Cardiovascular health, Risk stratification

## Abstract

**Aims:**

Both Life’s essential 8 (LE8) and retinal photographs are closely related to cardiovascular diseases (CVDs). We aimed to develop a novel deep learning–based tool for CVD risk stratification, termed RetiLE8, by predicting LE8 based on retinal photographs.

**Methods and results:**

This study was based on the UK Biobank, a prospective cohort study. Retinal photographs from the UK Biobank were used to train a deep learning model to predict LE8 scores, generating the RetiLE8 scores. Cox proportional hazards models were used to estimate the association of RetiLE8 with all-cause mortality, CVD mortality, and CVD events. Model performance was compared with that of LE8 and the Pooled Cohort Equations (PCE), a contemporary risk estimation tool, using Harrell’s concordance index (C-index) and continuous net reclassification improvement (NRI). Retinal photographs from 10 798 participants and 25 750 participants of the UK Biobank were utilized for the development and validation of RetiLE8, respectively. RetiLE8 showed a modest correlation with LE8. One standard deviation (SD) increase in the RetiLE8 score was associated with 13% (95% CI, 7–19%) lower all-cause mortality risk and 10% (4–14%) lower CVD event risk, independent of the LE8 score and covariates. The RetiLE8 score showed similar discrimination to the LE8 score and the PCE in predicting outcomes and significantly improved risk stratification beyond both tools.

**Conclusion:**

The RetiLE8 score may serve as a complementary tool for CVD risk stratification with existing tools. Prospective studies implementing this tool in clinical practice are warranted to evaluate its utility in real-world settings.

## Graphical Abstract

**Graphical Abstract:**
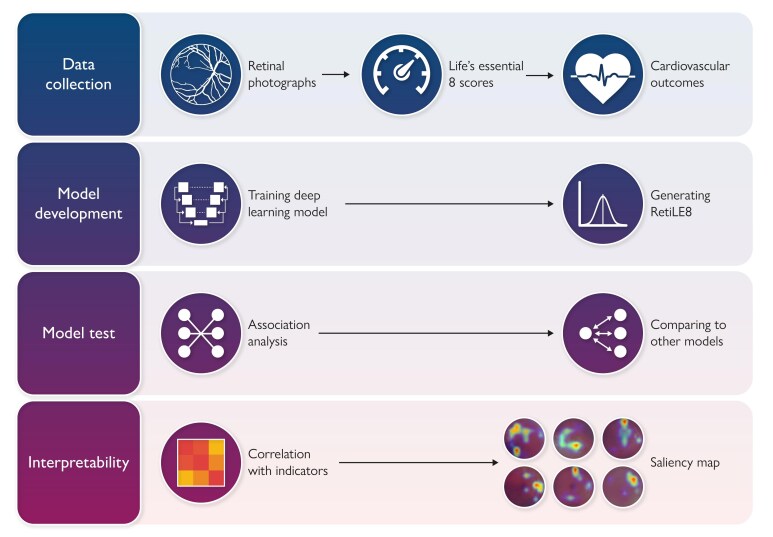

## Introduction

Cardiovascular diseases (CVDs) encompass a broad spectrum of diseases that affect the heart and vasculature, remaining the leading cause of mortality globally.^1^ The burden of CVDs is profound, involving life-years lost, diminished quality of life, and significant direct and indirect healthcare costs.^2,3^ The underlying pathological processes leading to CVDs often begin years before the onset of acute events or a clinical diagnosis,^4^ which provides opportunities for early detection or prevention.^5^ Thus, it is crucial to develop methods for effective CVD risk stratification.^6,7^

To enhance cardiovascular health (CVH) and facilitate its measurement and monitoring, the American Heart Association (AHA) published a scoring algorithm, the Life’s Simple 7 (LS7) in 2010,^2^ and a more updated algorithm of the Life’s Essential 8 (LE8) score in 2022.^8^ Having a higher LE8 score was associated with a broad range of health outcomes, including lower risk of CVDs, cancers, diabetes, dementia, and longer life expectancy.^9–11^ In addition, the Pooled Cohort Equation (PCE) is widely used to provide CVD risk stratification based on traditional clinical variables.^12^ Beyond these established clinical risk assessment frameworks, retinal imaging has attracted increasing attention as a source of imaging-derived biomarkers for systemic diseases.^13^ The retina is one of the most vascularized and metabolically active organs in the human body,^14^ allowing a range of systemic conditions, including CVDs, to manifest in the retina.^15–17^ With the rapid advancement of deep learning technology, recent studies have highlighted the effectiveness of retinal photographs for CVD risk stratification, due to their high accuracy in predicting specific CVDs and various associated risk factors.^18–20^ Multiple CVD risk factors, such as age, sex, and blood pressure, have been accurately predicted from retinal photographs using deep learning.^18,21^ The LE8 score was found to be associated with retinopathy and several retinal features such as the artery-to-vein ratio.^22–24^ However, evidence remains limited on whether LE8-related retinal features can serve as an additional metric to improve existing risk models.

We supposed that deep learning algorithms could extract LE8-related retinal features, as well as other traits associated with CVH, from retinal photographs. We therefore developed a deep learning algorithm to predict the LE8 score based on retinal photographs (termed Retinal LE8, RetiLE8) and evaluated its predictive performance and its added value when combined with existing risk estimation tools for risk stratification of death and CVD events. The RetiLE8 score is intended to complement the traditional LE8 score by incorporating CVD-relevant retinal information.

## Methods

### Study population

**Figure 1:**
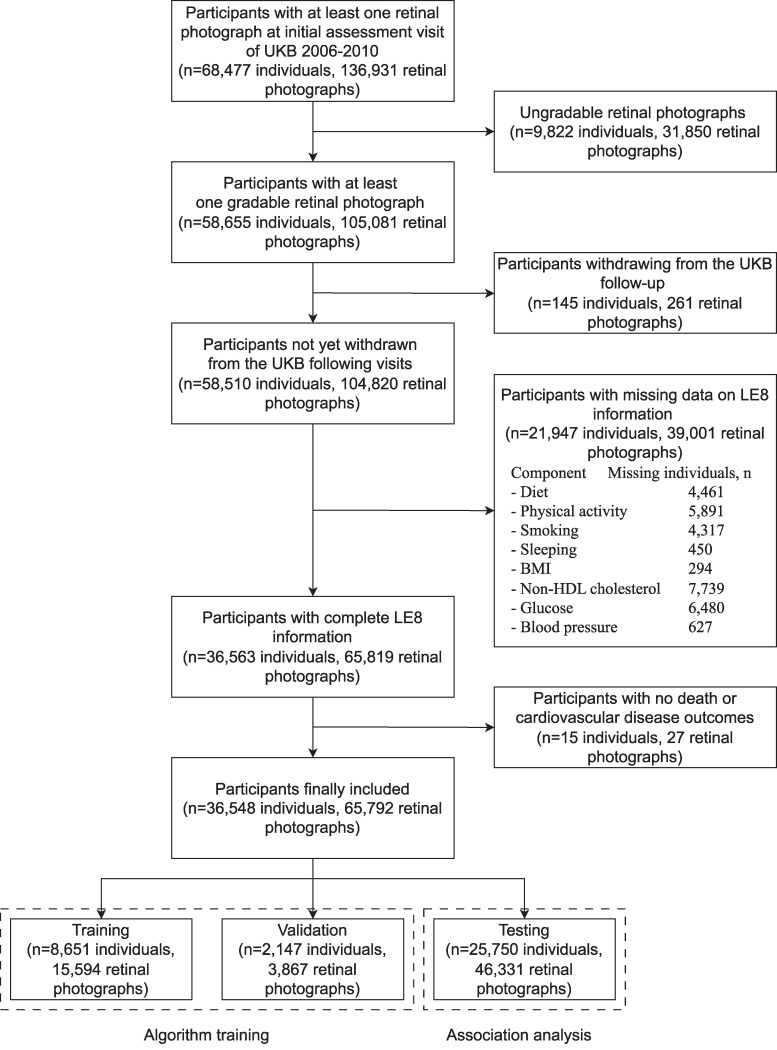
Flowchart of the sample selection.

We used data in the UK Biobank, a prospective cohort study in which about 500 000 participants aged 40–69 years across the UK were recruited between 2006 and 2010.^25^ The UK Biobank was approved by the National Information Governance Board for Health and Social Care and the National Health Service North West Centre for Research Ethics Committee (Ref: 11/NW/0382, 17 June 2011). All participants gave informed consent to participate and be followed up. The study was conducted in accordance with the principles of the Declaration of Helsinki. Each participant went through a series of questionnaires and health measurements, including retinal photography. Information on morbidity, mortality, and other health outcomes was collected. In this study, the participants with at least one retinal photograph available at the initial assessment of the UK Biobank (*n* = 68 477) were included. A quality control algorithm for retinal photographs was developed based on a deep learning algorithm, whose detailed information can be found in Supplementary material online, Method  *S1*. Participants without at least one gradable retinal photograph (*n* = 9822), withdrawing from the UK Biobank visit (*n* = 145), without complete information on the LE8 score (*n* = 21 947), and without death and CVD event outcomes (*n* = 15) were excluded. Finally, the analysis included 36 548 participants and 65 819 retinal photographs. The dataset was randomly divided into the following three independent datasets based on the individual level: 24% for training (*n* = 8651 individuals, 15 594 retinal photographs), 6% for validation (*n* = 2147 individuals, 3867 retinal photographs), and 70% for testing (*n* = 25 750 individuals, 46 331 retinal photographs). The training and validation datasets were used to develop the deep learning algorithm for the RetiLE8 score prediction, while the testing dataset was used to assess the associations between RetiLE8 and mortality and CVD events (*Figure 1*). To prevent model overfitting, we divided the dataset by individuals rather than by retinal photographs. As a result, multiple retinal photographs from the same individual, regardless of whether they were from the left or right eye, were kept within the same dataset and not distributed across the training, validation, and testing datasets.

### Calculation of the LE8 score

The LE8 score comprises eight CVH metrics (diet, physical activity, nicotine exposure, sleep duration, body-mass index [BMI], non–high-density lipoprotein [non-HDL] cholesterol, blood glucose, and blood pressure). In this study, we used the same method as a previous study to calculate the LE8 score based on UK Biobank variates.^26^ Consistent with previous UK Biobank studies,^26–28^ diet was defined using a more recent criterion for the ideal intake of healthy and unhealthy dietary components for CVH, which aligns with the AHA guidelines (Supplementary material online, *Table S1*). Each of the 8 CVH metrics was scored, ranging from 0 to 100 points, and the LE8 score was calculated as the mean of the eight metrics (Supplementary material online, *Table S2*).

### Prediction of the RetiLE8 score

A deep learning algorithm was trained to predict the LE8 score (ground truth) based on the retinal photographs utilizing training and validation datasets. The characteristics of the training and validation datasets are shown in Supplementary material online, *Table S3*. The retinal photographs were taken during the initial assessment visit of the UK Biobank 2006–2010. Captured without pupil dilation, the photographs were obtained using the Topcon 3D OCT-1000 Mark II (Topcon Corporation).

Full details of the training process of the deep learning algorithm to predict the LE8 score are provided in Supplementary material online, Method  *S2*. In brief, the ResNet50 architecture was used. The model was trained for 100 epochs on the training dataset, and the best-performing version on the validation dataset after a certain epoch was selected. The trained model was then used to predict RetiLE8 scores for the testing dataset to facilitate further association assessment. For participants with multiple gradable retinal photographs, the RetiLE8 scores were derived by averaging the predicted scores from all their retinal photographs.

### Outcomes

The outcomes of this study were all-cause mortality, CVD mortality, and CVD events. Hospitalization and mortality data from the National Health Service (NHS) registers were used to assess these outcomes. The data on mortality were available up to 31 December 2022, whereas the data on hospitalization were available up to 31 October 2022. CVDs were defined based on the European Systematic Coronary Risk Evaluation (SCORE) clinical guidelines^29^ (International Classification of Diseases [ICD]-10 codes, I10–15, I44–51, I20–25, and I61–73) when assessing CVD mortality and CVD events. In addition, for the analysis, including CVD events, we further excluded the participants who had CVDs at baseline.

### Covariates

The covariates in this study included age (continuous), sex (male, female), annual household income (<18 000, 18 000–30 999, 31 000–51 999, 52 000–100 000, and >100 000 pounds), ethnicity (White, Black, South Asian, and Other), education level (high [college or university degree], intermediate [A levels/AS levels or equivalent, O levels/GCSEs or equivalent], and low [other qualifications or no quantifications]), and alcohol status (never, previous, and current). The frequency of missing data of covariates is displayed in Supplementary material online, *Table S4*. For missing covariates, continuous variables were imputed with the mean value, whereas categorical variables were assigned a distinct value outside the existing categories.

### Statistical analysis

In both the development dataset (training set and validation set) and the testing dataset, baseline characteristics of the study participants were presented as mean (SD) for continuous variables and as frequencies with percentages for categorical variables. A scatter plot of the LE8 and RetiLE8 scores was presented to illustrate their correlation. A histogram of the RetiLE8 scores was generated to demonstrate its distribution. The baseline characteristics of the participants stratified by quartiles of the RetiLE8 scores were also provided. To investigate the influence of medication use, baseline characteristics were stratified by the use of insulin, blood pressure medication, or cholesterol-lowering medication.

Cox proportional hazards models were used to estimate the hazard ratios (HRs) and corresponding 95% CIs for the associations of the RetiLE8 score as categorical and continuous variables with outcomes of all-cause mortality, CVD mortality, and CVD events. The RetiLE8 score was divided into four quartiles when treated as a categorical variable, and the Z-score was transformed when treated as a continuous variable. Three models were constructed: a crude model; a second model adjusted for age, sex, annual household income, ethnicity, education level, and alcohol status; and a third model further adjusted for the original LE8 score. The LE8 and RetiLE8 scores were dichotomized into low and high groups based on their respective medians, and their joint associations with outcomes were examined. Subgroup interactions of age, sex, annual household income, ethnicity, education level, and alcohol status in the associations of the RetiLE8 score with all the outcomes were assessed.

To assess the independent and incremental risk stratification capability of the RetiLE8 score, we calculated the Harrell’s concordance index, differences in Harrell’s concordance index, and the continuous net reclassification improvement (NRI) index to compare the discrimination and reclassification performance of the RetiLE8 score, the LE8 score, and their combination across all outcomes.^30,31^ As a representative of contemporary risk stratification tools for CVD,^12^ the PCE was also compared with the RetiLE8 score. The covariates were included in all the models. The model prediction statistics were calculated for the median duration of the follow-up. Sensitivity analyses were additionally performed, excluding participants receiving cholesterol-lowering medication.

Several approaches were employed for the interpretation of RetiLE8. Differences in the two subscales and eight individual components of the LE8 score, as well as age, were assessed between participants with high vs. low LE8 or RetiLE8 scores. High and low groups were defined based on the median of LE8 or RetiLE8. All variables were Z-score-transformed prior to comparison. Standardized mean differences (SMD) were calculated to quantify group contrasts, and Student’s *t*-tests were performed to evaluate statistical significance. Pearson’s correlation coefficients (r) were calculated to assess the associations of the LE8 and RetiLE8 scores with age, individual components of the LE8 score, and related variables. These correlations were further examined within tertiles of the LE8 and RetiLE8 scores, respectively. The gradient-weighted class activation mapping (Grad-CAM) technique was applied to generate saliency maps, highlighting the importance of different regions of the retinal photographs in predicting the RetiLE8 score.

Statistical analyses were performed using Python, version 3.10, and R statistical software, version 4.1.0. Two-sided *P* < 0.05 was considered statistically significant. The analyses were conducted in March, 2025.

## Results

The testing dataset included 46 331 retinal photographs from 25 750 UK Biobank participants. At baseline, the participants had a mean age of 56.38 years (SD 8.15), with 11 757 (45.7%) males and 13 993 (54.3%) females (*Table 1*). Throughout the course of a median follow-up of 12.66 years (IQR, 12.56–12.80), 1425 (5.5%) of the participants died from all causes, and 213 (0.8%) died from CVD. Among the participants free of CVD (*n* = 24 612) before the beginning of the follow-up, 2428 (9.9%) had developed CVD during a median follow-up of 12.47 years (IQR 12.36–12.62). The correlation between LE8 and RetiLE8 was modest, with a Pearson’s correlation coefficient of 0.28. The mean absolute error was 8.51 (95% CI, 8.43–8.58), whereas the root mean squared error was 10.68 (95% CI, 8.68–12.69) (Supplementary material online, *Figure S2*). The distribution of the RetiLE8 score was presented in Supplementary material online, *Figure S3*. Differences in baseline characteristics across quartiles of the RetiLE8 score are summarized in Supplementary material online, *Table S5*. Both the LE8 score and the RetiLE8 score were lower among participants using insulin, blood pressure-lowering medication, or cholesterol-lowering medication (Supplementary material online, *Table S6*).

**Table 1:** Characteristics of the test set at initial assessment of UK Biobank

Variables	*n* = 25 750
Age [mean (SD)], years	56.38 (8.15)
Sex	
Male	11 757 (45.7)
Female	13 993 (54.3)
Annual household income, pounds	
<18 000	3937 (17.1)
18 000–30 999	5447 (23.7)
31 000–51 999	6159 (26.8)
52 000–100 000	5521 (24.0)
>100 000	1917 (8.3)
Ethnicity	
White	23 985 (93.4)
Black	510 (2.0)
South Asian	611 (2.4)
Other	576 (2.2)
Education level	
Low	7020 (27.4)
Intermediate	8573 (33.5)
High	10 019 (39.1)
Alcohol status	
Never	1023 (4.0)
Previous	786 (3.1)
Current	23 932 (93.0)
BMI [mean (SD)], kg/m^2^	27.17 (4.60)
Total cholesterol [mean (SD)], mg/dL	220.43 (43.25)
HDL cholesterol [mean (SD)], mg/dL	58.00 (15.10)
HbA1c [mean (SD)], %	5.40 (0.54)
Systolic pressure [mean (SD)], mmHg	136.66 (18.25)
Diastolic pressure [mean (SD)], mmHg	81.70 (10.01)
Diabetes	
No	24 566 (95.4)
Yes	1184 (4.6)
Hypertension	
No	19 345 (75.1)
Yes	6405 (24.9)
High cholesterol	
No	22 612 (87.8)
Yes	3138 (12.2)
Insulin	
No	25 541 (99.2)
Yes	209 (0.8)
Blood pressure medication	
No	20 793 (80.7)
Yes	4957 (19.3)
Cholesterol lowering medication	
No	21 342 (82.9)
Yes	4408 (17.1)
LE8 [mean (SD)]	67.93 (11.13)
LE8 sub-scores [mean (SD)]	
Health behavior	71.97 (13.57)
Health factor	63.89 (16.50)
Diet	43.34 (18.72)
Physical Activity	76.40 (34.54)
Smoking	78.13 (30.34)
Sleeping	89.99 (17.76)
BMI	70.24 (27.97)
Non-HDL cholesterol	48.85 (28.85)
Glucose	91.90 (18.53)
Blood Pressure	44.56 (32.68)

Abbreviations: SD, standard deviation; LE8, Life’s Essential 8; BMI, body-mass index; HDL, high-density lipoprotein.

A higher LE8 score was significantly associated with a lower risk of all-cause mortality, CVD mortality, and CVD events in each model (Supplementary material online, *Table S7*). After adjustment for all covariates and additionally for the LE8 score, the HR for all-cause mortality in the fourth quartile of the RetiLE8 score was 0.66 (95% CI, 0.53–0.81; *P* < 0.001) compared to the lowest quartile. One SD increase in the RetiLE8 score was associated with a HR of 0.87 (95% CI, 0.81–0.93; *P* < 0.001) for all-cause mortality. For CVD mortality, the HR for the fourth quartile of the RetiLE8 score was 0.61 (95% CI, 0.33–1.12; *P* = 0.11) compared to the lowest quartile, whereas one SD increase in the RetiLE8 score was associated with a HR of 0.86 (95% CI, 0.70–1.05; *P* = 0.13). For CVD events, the HR for the fourth quartile of the RetiLE8 score was 0.80 (95% CI, 0.69–0.92; *P* = 0.002) compared to the lowest quartile, and each one SD increase in the RetiLE8 score was associated with a HR of 0.90 (95% CI, 0.86–0.96; *P* < 0.001) (*Table 2*). Compared to participants with both low LE8 and low RetiLE8 scores, those with both high LE8 and high RetiLE8 scores had a lower risk of all-cause mortality (HR, 0.57; 95% CI, 0.48–0.68; *P* < 0.001), CVD mortality (HR, 0.36; 95% CI, 0.21–0.60; *P* < 0.001), and CVD events (HR, 0.60; 95% CI, 0.53–0.68; *P* < 0.001) (Supplementary material online, *Table S8*).

**Table 2:** Associations of RetiLE8 with all-cause mortality, cardiovascular mortality, and cardiovascular disease events

Outcomes	RetiLE8	n/*n*	LE8 Mean(Range)	RetiLE8 Mean(Range)	Crude model^a^		Adjusted model 1^b^		Adjusted model 2^c^	
					HR(95% CI)	*P*	HR(95% CI)	*P*	HR(95% CI)	*P*
All-cause mortality	Q1	576/6438	64.42(18.75–97.50)	64.72(59.48–65.98)	1.00(ref)		1.00(ref)		1.00(ref)	
	Q2	424/6437	66.41(26.25–97.50)	66.93(65.98–67.91)	0.73(0.64,0.82)	<0.001	0.92(0.81,1.04)	0.18	0.95(0.83,1.07)	0.39
	Q3	297/6437	68.46(27.50–100.00)	69.04(67.91–70.36)	0.50(0.44,0.58)	<0.001	0.86(0.74,0.99)	0.04	0.91(0.78,1.05)	0.18
	Q4	128/6438	72.42(24.38–97.50)	72.93(70.36–84.41)	0.21(0.18,0.26)	<0.001	0.59(0.48,0.72)	<0.001	0.66(0.53,0.81)	<0.001
	Per 1-SD increase ^d^				0.56(0.53,0.60)	<0.001	0.83(0.77,0.89)	<0.001	0.87(0.81,0.93)	<0.001
Cardiovascular disease mortality	Q1	96/6438	64.42(18.75–97.50)	64.72(59.48–65.98)	1.00(ref)		1.00(ref)		1.00(ref)	
	Q2	68/6437	66.41(26.25–97.50)	66.93(65.98–67.91)	0.70(0.51,0.95)	0.02	0.96(0.70,1.32)	0.80	1.03(0.75,1.42)	0.84
	Q3	36/6437	68.46(27.50–100.00)	69.04(67.91–70.36)	0.37(0.25,0.54)	<0.001	0.74(0.50,1.09)	0.13	0.84(0.56,1.25)	0.38
	Q4	13/6438	72.42(24.38–97.50)	72.93(70.36–84.41)	0.13(0.07,0.23)	<0.001	0.48(0.26,0.89)	0.02	0.61(0.33,1.12)	0.11
	Per 1-SD increase ^d^				0.47(0.39,0.56)	<0.001	0.78(0.64,0.95)	0.01	0.86(0.70,1.05)	0.13
Cardiovascular disease events	Q1	938/6012	64.54(18.75–97.50)	64.73(59.48–65.98)	1.00(ref)		1.00(ref)		1.00(ref)	
	Q2	664/6125	66.52(26.25–97.50)	66.93(65.98–67.91)	0.67(0.61,0.74)	<0.001	0.82(0.74,0.91)	<0.001	0.85(0.77,0.94)	0.002
	Q3	500/6168	68.61(27.50–100.00)	69.05(67.91–70.36)	0.49(0.44,0.55)	<0.001	0.78(0.69,0.87)	<0.001	0.83(0.74,0.93)	0.002
	Q4	326/6307	72.52(24.38–97.50)	72.94(70.36–84.41)	0.30(0.27,0.35)	<0.001	0.70(0.61,0.80)	<0.001	0.80(0.69,0.92)	0.002
	Per 1-SD increase ^d^				0.62(0.59,0.65)	<0.001	0.86(0.81,0.90)	<0.001	0.90(0.86,0.96)	<0.001

Abbreviations: LE8, Life’s Essential 8; SD, standard deviation; HR, hazard ratio; Q1-Q4, quartile 1- quartile 4.

^a^Crude models.

^b^Adjusted for age, sex, annual household income, ethnicity, education level, and alcohol status.

^c^Adjusted for age, sex, annual household income, ethnicity, education level, alcohol status, and the LE8 score (continuous).

^d^RetiLE8 scores were Z-score transformed to assess the association per 1-SD increase with the outcome.

Subgroup analyses showed significant interactions between the RetiLE8 score and age and education subgroups with the risk of all-cause mortality (Supplementary material online, *Figure S4*). The association of the RetiLE8 score with all-cause mortality was stronger in the younger group (*P* for interaction = 0.05) and among individuals with a lower education level (*P* for interaction = 0.04). No significant interactions were observed between the RetiLE8 score and covariate subgroups in relation to the risk of CVD mortality (Supplementary material online, *Figure S5*). Significant interactions were observed between the RetiLE8 score and age and sex subgroups in relation to the risk of CVD events (Supplementary material online, *Figure S6*). The association between the RetiLE8 score and CVD events was stronger in younger individuals (*P* for interaction = 0.01) and in females (*P* for interaction= 0.003).

The concordance index for the RetiLE8 score was 0.727 (95% CI, 0.715–0.740) for all-cause mortality, 0.810 (95% CI, 0.783–0.836) for CVD mortality, and 0.703 (95% CI, 0.693–0.713) for CVD events. The RetiLE8 score demonstrated a slightly reduced performance in predicting outcomes compared with the LE8 score, whereas its combination with LE8 demonstrated improved predictive performance (*Table 3*). Compared with the LE8 score, the RetiLE8 score yielded significant lower concordance indices for predicting CVD mortality (ΔC index, −0.011; 95% CI, −0.021, −0.001; *P* = 0.03) and CVD events (ΔC index, −0.006; 95% CI, −0.009, −0.003; *P* < 0.001), and significant lower NRI for predicting CVD events (NRI, −6.3%; 95% CI, −10.3%, −2.3%; *P* = 0.006). Relative to the LE8 score alone, adding the RetiLE8 score resulted in higher concordance indices and NRIs for predicting both all-cause mortality (ΔC index, 0.002; 95% CI, 0.000, 0.003; *P* = 0.02; NRI, 4.9%; 95% CI, 0.1%, 9.6%; *P* = 0.04) and CVD events (ΔC index, 0.001; 95% CI, 0.000, 0.002; *P* = 0.02; NRI, 3.9%; 95% CI, 0.2%, 7.7%; *P* = 0.04). The RetiLE8 score and the PCE demonstrated similar performance in predicting the outcomes, and its combination with PCE demonstrated improved predictive performance (Supplementary material online, *Table S9*). Compared with the PCE, the RetiLE8 score yielded significant higher NRIs for predicting all-cause mortality (NRI, 6.4%; 95% CI, 0.9–10.1%; *P* = 0.02) and CVD events (NRI, 5.2%; 95% CI, 2.1–8.9%; *P* = 0.006), but a significant lower concordance index for predicting CVD events (ΔC index, −0.003; 95% CI, −0.005, −0.001; *P* = 0.008). Adding the RetiLE8 score to the models, including the PCE, resulted in a significant but minor improvement in prediction for all-cause mortality and CVD events, while no improvement in CVD mortality prediction was observed. After excluding participants receiving cholesterol-lowering medication, the results remained materially unchanged (Supplementary material online, *Table S10*, *Table S11*).

**Table 3:** Comparison of discrimination and reclassification among the life’s essential 8, RetiLE8, and their combination

Outcomes	Model	Concordance(95% CI)	ΔC index(95% CI)	*P*	NRI(95% CI)	*P*
All-cause mortality	LE8	0.731(0.718,0.743)	0(ref)		0(ref)	
	RetiLE8	0.727(0.715,0.740)	−0.003(−0.007,0.000)	0.08	−4.5%(−9.3%,0.8%)	0.12
	LE8 + RetiLE8	0.732(0.720,0.745)	0.002(0.000,0.003)	0.02	4.9%(0.1%,9.6%)	0.04
Cardiovascular disease mortality	LE8	0.821(0.796,0.846)	0(ref)		0(ref)	
	RetiLE8	0.810(0.783,0.836)	−0.011(−0.021,−0.001)	0.03	−11.3%(−22.1%,3.6%)	0.12
	LE8 + RetiLE8	0.822(0.797,0.847)	0.001(−0.001,0.003)	0.27	6.2%(−4.5%,17.1%)	0.27
Cardiovascular disease events	LE8	0.709(0.700,0.719)	0(ref)		0(ref)	
	RetiLE8	0.703(0.693,0.713)	−0.006(−0.009,−0.003)	<0.001	−6.3%(−10.3%,−2.3%)	0.006
	LE8 + RetiLE8	0.711(0.701,0.720)	0.001(0.000,0.002)	0.02	3.9%(0.2%,7.7%)	0.04

All models included age, sex, annual household income, ethnicity, education level, and alcohol status. LE8, Life’s Essential 8; ΔC index, difference in the Harrell’s concordance index; NRI, net reclassification improvement index.

**Figure 2:**
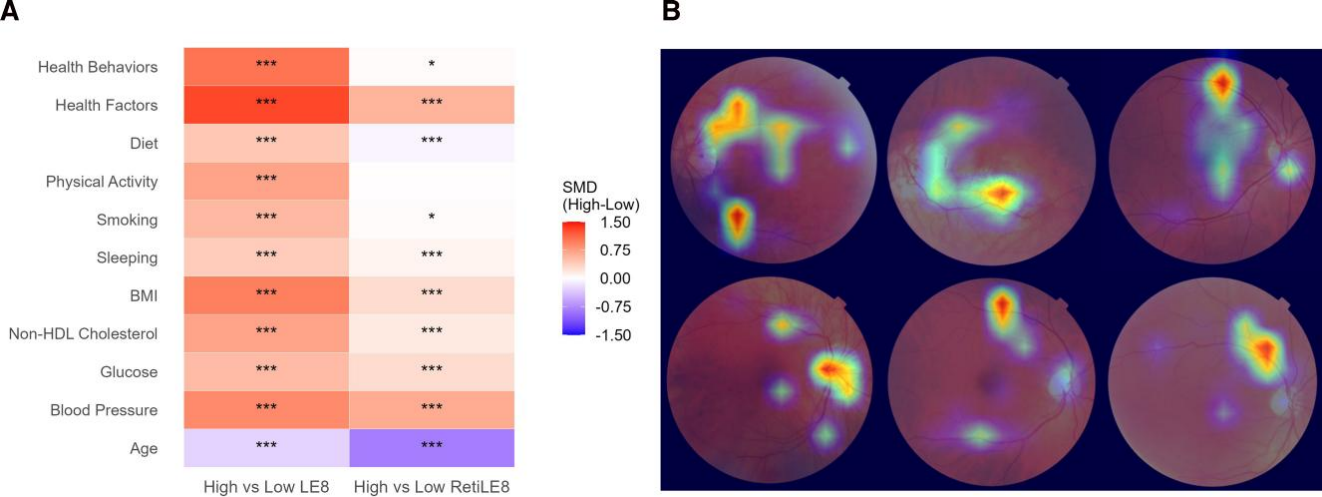
Interpretability of RetiLE8: correlations with other factors and saliency maps. (*A*) The heatmap displays standardized differences between participants with low vs. high LE8 or RetiLE8 scores. Group stratification was based on the median of LE8 or RetiLE8. All variables were Z-score transformed before comparison. Standardized mean differences (SMD) were calculated to quantify group contrasts, and Student’s t-tests were used to assess statistical significance. Significance levels are indicated as: *P < 0.05, ** *P* < 0.01, *** *P* < 0.001. (*B*) Saliency map generated using Grad-CAM. Warmer regions indicate areas to which the deep learning model assigned greater importance when predicting the RetiLE8 scores.

Significant differences across the two subscales, eight individual components of the LE8 score, and age were observed between participants with high and low RetiLE8 scores, except for physical activity (*Figure 2A*). Larger standardized mean differences were noted for health factors and age than for health behaviours. The RetiLE8 score showed stronger correlations with health factors and age than with health behaviours (Supplementary material online, *Table S12*). Both the LE8 and RetiLE8 scores demonstrated stronger correlations with age, individual components of the LE8 score, and related variables in the highest tertile (Supplementary material online, *Table S13*, *Table S14*). Saliency maps indicated that RetiLE8 mostly focuses on the macula, optic disc, and retinal vessels (*Figure 2B*).

## Discussion

In this study, the RetiLE8 score was developed by training a deep learning algorithm to predict the LE8 score based on retinal photographs. The RetiLE8 score was significantly associated with all-cause mortality and CVD-related outcomes. Individuals in the 4th quartile of the RetiLE8 score had a lower risk of all-cause mortality, CVD mortality, and CVD events compared to those in quartile 1, after adjusting for confounders. The RetiLE8 score demonstrated CVD risk stratification performance similar to that of the LE8 score and the PCE. In addition to the PCE or the LE8 score, the RetiLE8 score provided a significant improvement in predicting all-cause mortality and CVD events.

Research has demonstrated that retinal vasculature and other structures observed in retinal photographs are closely associated with CVD and its risk factors.^18,19,21,32^ One study segmented the retinal vasculature from retinal photographs to calculate vascular density and fractal dimension, two key indicators of retinal vascular. Strong associations were found between these measures and both blood pressure and new-onset hypertensive heart disease.^32^ Deep learning algorithms have also been applied to retinal photographs to predict CVD risk factors, including age, sex, and blood pressure, achieving reasonable accuracy.^18,21^ In another study, retinal photographs were analysed using a deep learning algorithm to estimate coronary artery calcium scores, showing strong potential for CVD risk stratification.^19^ To the best of our knowledge, this is the first study to predict mortality and CVH outcomes by deriving the LE8 score from retinal photographs.

Previous studies have separately examined the associations between the retina and each of the eight components of LE8.^18,21,24,32–34^ The associations of the four health behaviours within the LE8 framework, alongside other lifestyle factors including sedentary behaviour and screen time, with retinal features have been investigated in several studies. Significant correlations between lifestyle factors and annotated retinal characteristics were revealed.^24,33,34^ In particular, unhealthy lifestyle patterns have been linked to reduced retinal vessel calibre and higher risk of age-related macular degeneration and retinal vascular occlusion.^24^ Moreover, alterations in vascular complexity, such as reduced vessel density and lower fractal dimension, have been linked to elevated risks of hypertension and diabetes.^32^ Beyond these associations with observable retinal features, all four health factors of LE8 have been predicted in prior studies using retinal photographs with deep learning algorithms. Among these, blood pressure has shown the strongest agreement between predicted and measured values, whereas the predictive performance for HbA1c, BMI, and cholesterol was comparatively modest.^18,21^ Expanding on these findings, our study investigates the association between retinal features and CVD risk factors considered collectively under the LE8 framework, as an approach not adopted in prior research. The present research further highlights the strong relationship between retina and CVH, and the potential of retinal photographs for CVD risk stratification.

A significant but modest correlation was observed between the RetiLE8 score and the original LE8 score. This is expected for several reasons. First, RetiLE8 is a prediction-based surrogate derived solely from retinal photographs. The extent to which individual LE8 metrics are manifested in retinal structure and vasculature varies substantially, which affects the model’s ability to detect corresponding retinal features. This is consistent with findings from previous works showing large heterogeneity in the predictive accuracy of different systemic traits using retinal imaging.^18,21^ In our study, the correlations between RetiLE8 and individual LE8 metrics also varied. For example, blood pressure, which is known to have a strong influence on the retina, showed the strongest correlation, whereas metrics relying on self-reported questionnaires, such as diet and physical activity, had weaker correlations, likely due to measurement error and shorter-term biological influence. Second, RetiLE8 may capture aspects of cardiovascular health that extend beyond the LE8 framework. Age, for instance, is a major CVD risk factor and can be predicted from retinal photographs with high accuracy. The moderate correlation reflects both physiological relevance and measurement characteristics of RetiLE8 as a surrogate imaging biomarker, indicating that it provides complementary rather than identical information to the LE8 score.

Although clear and strong associations were observed between LE8 scores and their own components, the differences became weaker when stratified by RetiLE8 scores. This attenuation can be interpreted from several perspectives. First, RetiLE8 is a prediction-based measure derived from retinal photographs, and therefore, it inevitably captures only part of the information contained in the original LE8 score. Prediction error and the variability in how different LE8 components manifest in retinal structure may dilute these associations. Second, health factors, such as blood pressure, have long-term and cumulative effects on the retina, whereas health behaviours, such as physical activity, may not lead to immediate or visible retinal alterations. In our study, both retinal photographs and LE8 metrics were collected at baseline, and the duration of participants’ current lifestyle status is unknown. Short-term changes in health behaviours may not yet be reflected in retinal phenotypes. Third, several LE8 behaviour components rely on self-reported questionnaires, which may introduce misclassification and measurement bias. Taken together, the relatively weaker correlations with RetiLE8 appear biologically and methodologically plausible. Longitudinal studies are needed to further investigate the time-dependent effects of LE8 components on retinal appearance.

Despite the modest correlation, the RetiLE8 score appears to hold clinical relevance. We thought that the algorithm may have extracted retinal features related to CVH beyond those directly captured by the LE8 metrics. Multiple retinal features, such as vessel density, fractal dimension, and calibre, are associated with CVD.^32,35^ Although the algorithm was not trained to predict CVD outcomes directly, the LE8 score, as a stable measure of CVH, may have enabled the algorithm to identify additional relevant features. In our study, the algorithm primarily focused on CVH-related regions, including the macula, optic disc, and retinal vessels, when generating the RetiLE8 score, supporting its biological plausibility in CVD risk stratification. Besides, adding the RetiLE8 score to the original LE8 score or the PCE resulted in a modest but significant improvement in discrimination and classification for predicting all-cause mortality and CVD events. This finding further suggests the possible existence of additional features beyond the LE8 score.

Significant interaction effects were observed between the RetiLE8 score and several biological risk factors, including sex and age, on all-cause mortality and CVD events. Sex is a major risk factor for CVD, and sex hormones are believed to play a complex role in multiple mechanisms contributing to CVD risk.^36^ Deep learning algorithms can also predict sex from retinal photographs, indicating the presence of sex-specific retinal features.^37^ Previous studies have shown that female sex hormones can exacerbate retinal neurodegeneration.^38^ We speculate that CVD-related changes in the retina may manifest earlier in females, which could explain why the association between the RetiLE8 score and CVD events was stronger in women. We also observed that the associations of the RetiLE8 score with all-cause mortality and CVD events were weaker in older participants. This finding is consistent with a previous study in which models predicting age or systolic blood pressure based on retinal microvascular function performed better in younger individuals.^39^ A plausible explanation is that the accumulation of physiological and structural changes with aging may obscure CVH-related retinal features, thereby reducing the reliability of RetiLE8 in older populations.

Although education is not a biological risk factor, a significant interaction was observed between the RetiLE8 score and education on all-cause mortality. First, lower education has been associated with multiple adverse health outcomes, such as diabesity.^40^ Socioeconomic deprivation may play an important role in this interaction. Second, previous studies have reported significant interactions between self-rated health and education on all-cause mortality.^41^ Because several metrics of the original LE8 score are derived from self-reported questionnaires, the predicted RetiLE8 score could potentially be influenced by participants’ educational attainment. Finally, education has been linked to several retinal conditions and features, including age-related macular degeneration and macular pigment,^42,43^ which could also contribute to the observed interaction.

The results of our study must be seen in light of some limitations. First, the UK Biobank cohort is predominantly composed of non-Hispanic White participants, which limits the generalizability of our findings. Future studies in populations with more diverse ethnic and regional backgrounds are warranted. Second, no external validation was performed in the current study, and future work is needed to confirm the performance of the developed algorithm in independent cohorts. Third, because UK Biobank participants generally have higher socioeconomic status, selection bias may have been introduced. Fourth, the correlation between the original LE8 score and the RetiLE8 score was modest. Although the RetiLE8 score was significantly associated with CVD-related outcomes, the limited interpretability of deep learning algorithms may restrict widespread clinical adoption. Finally, all ungradable retinal photographs were identified using a quality control model and removed; however, a significant portion of the photographs remained of poor quality. Real-world validation is needed before the algorithm can be clinically implemented.

## Conclusion

In this study, we developed and validated a novel complementary metric for CVD risk stratification, the RetiLE8 score, using a deep learning algorithm trained to predict the LE8 score from retinal photographs. The modest correlation between the RetiLE8 score and the LE8 score indicates that RetiLE8 is not a substitute for LE8 but rather captures complementary, imaging-derived aspects of cardiovascular health. Our findings demonstrate that RetiLE8 is strongly associated with all-cause mortality and CVD events, independent of confounders and the original LE8 score. The RetiLE8 score holds promise as a complementary imaging-based marker to existing risk stratification tools. Prospective studies implementing this tool in clinical practice are warranted to evaluate its utility in real-world settings.

## Supplementary Material

ztag041_Supplementary_Data

## Data Availability

All data used in this study are available from the UK Biobank upon approved application (http://www.ukbiobank.ac.uk/register-apply). Relevant R and python code is available from the corresponding author upon reasonable request.
