# Supplementary material for: Retinal photographs to predict life’s essential 8 for cardiovascular risk stratification: a novel deep-learning-based tool
Source: Eur Heart J Digit Health. 2026 Mar 6;7(3):ztag041. doi: 10.1093/ehjdh/ztag041 (PMC13034666; doi:10.1093/ehjdh/ztag041)
Supplement: ztag041_Supplementary_Data [file ztag041_supplementary_data.docx]

**Supplemental Material**

**Content**

**Supplemental Method 1.** Development of the quality control model for retinal photographs.

**Supplemental Method 2.** Development of the model for RetiLE8 prediction.

**Table S1.** Calculation of the diet factor of the Life’s Essential 8 score.

**Table S2.** Calculation of the Life’s Essential 8 score.

**Table S3.** Characteristics of the training and validation dataset.

**Table S4.** Frequency of Missing Data of Covariates.

**Table S5.** Characteristics of the test set at initial assessment of UK Biobank stratified by the RetiLE8 score.

**Table S6.** Characteristics of the test set at initial assessment of UK Biobank stratified by medication use.

**Table S7.** Associations of Life’s Essential 8 with all-cause mortality, cardiovascular mortality and cardiovascular disease events.

**Table S8.** Joint associations of Life’s Essential 8 and RetiLE8 with all-cause mortality, cardiovascular mortality and cardiovascular disease events.

**Table S9.** Comparison of discrimination and reclassification among pooled cohort equations, RetiLE8, and their combination.

**Table S10.** Comparison of discrimination and reclassification among the Life’s Essential 8, RetiLE8, and their combination excluding participants receiving cholesterol lowering medication.

**Table S11.** Comparison of discrimination and reclassification among pooled cohort equations, RetiLE8, and their combination excluding participants receiving cholesterol lowering medication.

**Table S12.** Pearson’s correlations of LE8 and RetiLE8 scores with age, individual LE8 components, and related variables.

**Table S13.** Pearson’s correlations of LE8 scores with age, individual LE8 components, and related variables stratified by LE8 tertiles.

**Table S14.** Pearson’s correlations of RetiLE8 scores with age, individual LE8 components, and related variables stratified by RetiLE8 tertiles.

**Figure S1.** Receiver operating characteristic curve of the retinal photograph quality control model.

**Figure S2.** Correlations between the observed Life’s Essential 8 and the predicted Retinal Life’s Essential 8 (RetiLE8).

**Figure S3.** Distribution of Retinal Life’s Essential 8 (RetiLE8).

**Figure S4.** Subgroup analysis for the associations between per RetiLE8 1-SD increase and all-cause mortality.

**Figure S5.** Subgroup analysis for the associations between per RetiLE8 1-SD increase and cardiovascular mortality.

**Figure S6.** Subgroup analysis for the associations between per RetiLE8 1-SD increase and cardiovascular disease events.

**Supplemental Method 1.** Development of the quality control model for retinal photographs.

A deep learning algorithm was developed to automatically identify ungradable retinal photographs. A sample of 2,000 retinal photographs from the UK Biobank was used. The images were labeled as gradable or ungradable based on presence or absence of motion artifact, media opacity, and ability to clearly distinguish retinal vessels on the photographs. The images were down-sampled to achieve a balanced number across groups, and split into training, validation and testing datasets in a 3:2:5 ratio. The images were center-cropped to 1456×1456, resized to 320×320, and randomly flipped both vertically and horizontally with a probability of 0.5.

The ResNet50 architecture was used. The input image first passes through a 7×7 convolutional layer with a stride of 2, which produces 64 feature maps, followed by a 3×3 max pooling layer that reduces the spatial dimensions. Then, the network is divided into four stages of residual blocks, and each of them consists of a 1×1 convolution, a 3×3 convolution, and another 1×1 convolution, with batch normalization and ReLU activation, along with identity or projection shortcuts to preserve gradient flow. The four stages contain 3, 4, 6 and 3 residual blocks, respectively, producing 256, 512, 1024, and 2048 feature maps. After these stages, global average pooling is applied to the 2048 feature maps, resulting in a 2048-dimensional feature vector. This vector is then fed into a fully connected layer that outputs two values for classification. Cross entropy loss was utilized as the loss function. Adam optimizer with a learning rate of 1e-03 was used. The model was trained for 100 epochs on the training dataset, and the version with the highest accuracy on the validation dataset after a certain epoch was selected.

The selected model achieved an accuracy of 91.3%, a sensitivity of 91.9%, a specificity of 90.7%, and an area under the receiver operating characteristic curve (AUC) of 0.972 on the testing dataset (**Figure S1**). The model was then applied on the retinal photographs collected at initial assessment visit of UK Biobank 2006-2010, resulting in removal of 23.3% of the original images (**Figure 1**). The removal rate is similar to other published studies of UK Biobank retinal photographs.

**Supplemental Method 2.** Development of the model for RetiLE8 prediction.

The LE8 score was first Z-score transformed to accelerate the model convergence during the training process. The retinal photographs were center-cropped to 1456×1456, resized to 320×320, and randomly flipped both vertically and horizontally with a probability of 0.5 when training. ResNet50 architecture was utilized, whose architecture has been detailed in Supplemental Method 1. Specifically, the fully connected layer outputs a single value for this regression task. Mean squared error (MSE) was utilized as the loss function. Adam optimizer with a learning rate of 1e-03 was used. The model was trained for 100 epochs on the training dataset, and the version with the highest accuracy on the validation dataset after a certain epoch was selected. When applying the algorithm to the testing dataset, the predicted RetiLE8 scores were inverse Z-score transformed using the same mean and standard deviation from the training dataset to revert to the original scale.

**Table S1.** Calculation of the diet factor of the Life’s Essential 8 score.

| Diet component | Intake goal | Field IDs | Amount per serving |
| --- | --- | --- | --- |
| Fruit | 3 servings/day | 1309 (pieces fresh fruit/day)  1319 (pieces dried fruit/day) | 1309 – 1 piece  1319 – 5 pieces |
| Vegetable | 3 servings/day | 1289 (tablespoons cooked vegetables/day)  1299 (salad/raw vegetables/day) | 3 heaped tablespoons |
| Whole grains | 3 servings/day | 1438, 1448 (whole meal/wholegrain bread slices/week)  1458, 1468 (bran/oat/muesli cereal bowls/week) | 1438/1448 – 1 slice/day  1458/1468 – 1 bowl/day |
| (Shell)fish | ≥2 servings/week | 1329 (oily fish/week)  1339 (non-oily fish/week) | Once/week |
| Dairy | 2 servings/day | 1408 (cheese/week)  1418 (milk type) | 1408 – 1 piece/day  1418 – 1 glass/day if consumption of any type of milk |
| Vegetable oils | 2 servings/day | 1428 (Flora Pro-Active/Benecol spread)  2654 (Flora Pro-Active/Benecol, soft margarine -, olive oil based -, polyunsaturated/sunflower oil based -, other low/reduced fat spread)  1438 (bread slices/week) | 1 serving/day if in combination with eating at least 2 slices of bread (ID 1438) |
| Refined grains | ≤2 servings/day | 1438, 1448 (white, brown, other bread slices/week)  1458, 1468 (biscuit, other cereals/week) | 1438/1448 – 1 slice/day  1458/1468 – 1 bowl/day |
| Processed meats | ≤1 serving/week | 1349 (processed meat/week or daily)  3680 (age when last ate meat) | 1349 – 1 piece/day  3680 – 0 pieces/day if indicated having never eaten meat |
| Unprocessed meats | ≤2 servings/week | 1359 (poultry/week or day)  1369 (beef/week or day)  1379 (lamb or mutton/week or day)  1389 (pork/week or day)  3680 (age when last ate meat) | 1359-1389 – once/week  3680 – 0 pieces/day if indicated having never eaten meat |
| Sugar-sweetened beverages | Don’t drink | 6144 (never consumes drinks containing sugar) | Only 0 servings were possible here. |

Field IDs and serving sizes used per diet component in UK Biobank with available data from the general baseline questionnaire. If participants achieved the intake goal, they were considered to have an adequate intake of the diet component.

Scoring criteria for dietary recommendations for cardiovascular health: 1: If intake goal met; 0: If intake goal not met. (Range: 0-10)

**Table S2.** Calculation of the Life’s Essential 8 score.

| LE8 metric | Method of measurement | Field IDs | Quantification of LE8 metric |
| --- | --- | --- | --- |
| Diet | Measurement: A more recent definition of ideal intake of dietary components for cardiovascular health, detailed in Table S1 | Table S1 | Scoring:  Points Diets score (points)  100 8–10  80 6–7  50 4–5  25 2–3  0 0–1 |
| Physical activity (PA) | Self-reported minutes of moderate or vigorous PA per week | 884 (Number of days/week of moderate physical activity 10+ minutes)  894 (Duration of moderate activity)  904 (Number of days/week of vigorous physical activity 10+ minutes)  914 (Duration of vigorous activity) | Scoring:  Points Minutes  100 ≥150  90 120–149  80 90–119  60 60–89  40 30–59  20 1–29  0 0 |
| Tobacco/nicotine exposure | Self-reported use of cigarettes; or secondhand smoke exposure | 20116 (Smoking status)  1249 (Past tobacco smoking)  2897 (Age stopped smoking)  1259 (Smoking/smokers in household) | Scoring:  Points Status  100 Never smoker  75 Former smoker, quit ≥5 y  50 Former smoker, quit 1–<5 y  25 Former smoker, quit <1 y  0 Current smoker  Subtract 20 points (unless score is 0) for living with active indoor smoker in home |
| Sleep health | Self-reported average hours of sleep per night | 1160 (Sleep duration) | Scoring:  Points Level  100 7–<9  90 9–<10  70 6–<7  40 5–<6 or ≥10  20 4–<5  0 <4 |
| Body mass index | Measurement: Body weight (kilograms) divided by height squared (meters squared) | 21001 (Body mass index[BMI]) | Scoring:  Points Level  100 <25  70 25.0–29.9  30 30.0–34.9  15 35.0–39.9  0 ≥40.0 |
| Blood lipids (non-HDL cholesterol) | Measurement: Plasma total and HDL cholesterol with calculation of non–HDL cholesterol | 30690 (Cholesterol)  30760 (HDL cholesterol)  6153 (Medication for cholesterol, blood pressure, diabetes, or take exogenous hormones)  6177 (Medication for cholesterol, blood pressure or diabetes) | Metric:  Non–HDL cholesterol (mg/dL)  Scoring:  Points Level  100 <130  60 130–159  40 160–189  20 190–219  0 ≥220  If drug-treated level, subtract 20 points |
| Blood glucose | Measurement: HbA1c and history of Diabetes | 2443 (Diabetes diagnosed by doctor)  30750 (Glycated hemoglobin [HbA1c]) | Metric: HbA1c (%)  Scoring:  Points Level  100 No history of diabetes HbA1c <5.7  60 No diabetes and HbA1c 5.7–6.4  40 Diabetes with HbA1c <7.0  30 Diabetes with HbA1c 7.0–7.9  20 Diabetes with HbA1c 8.0–8.9  10 Diabetes with Hb A1c 9.0–9.9  0 Diabetes with HbA1c ≥10.0 |
| Blood pressure (BP) | Measurement: Appropriately measured systolic and diastolic blood pressures | 93 (Systolic blood pressure, manual reading)  4080 (Systolic blood pressure, automated reading)  94 (Diastolic blood pressure, manual reading)  4079 (Diastolic blood pressure, automated reading)  6153 (Medication for cholesterol, blood pressure, diabetes, or take exogenous hormones)  6177 (Medication for cholesterol, blood pressure or diabetes) | Metric: Systolic and diastolic BPs (mm Hg)  Scoring:  Points Level  100 <120 and <80  75 120–129 and <80  50 130–139 or 80–89  25 140–159 or 90–99  0 ≥160 or ≥100  Subtract 20 points if treated level |

**Table S3.** Characteristics of the training and validation dataset.

| Variables | Train | Validation |
| --- | --- | --- |
|  | N=8651 | N=2147 |
| Age (mean [SD]), years | 56.12 (8.23) | 56.40 (8.19) |
| Sex |  |  |
| Male | 3891 (45.0) | 993 (46.3) |
| Female | 4760 (55.0) | 1154 (53.7) |
| Annual household income, pounds |  |  |
| <18,000 | 1305 (16.9) | 348 (18.3) |
| 18,000-30,999 | 1799 (23.4) | 431 (22.7) |
| 31,000-51,999 | 2090 (27.1) | 510 (26.8) |
| 52,000-100,000 | 1875 (24.3) | 478 (25.2) |
| >100,000 | 634 (8.2) | 133 (7.0) |
| Ethnicity |  |  |
| White | 8020 (93.1) | 1970 (92.1) |
| Black | 155 (1.8) | 44 (2.1) |
| South Asian | 221 (2.6) | 64 (3.0) |
| Other | 219 (2.5) | 61 (2.9) |
| Education level |  |  |
| Low | 2276 (26.5) | 621 (29.1) |
| Intermediate | 2913 (33.9) | 691 (32.4) |
| High | 3414 (39.7) | 824 (38.6) |
| Alcohol status |  |  |
| Never | 367 (4.2) | 88 (4.1) |
| Previous | 247 (2.9) | 77 (3.6) |
| Current | 8034 (92.9) | 1982 (92.3) |
| BMI (mean [SD]), kg/m² | 27.11 (4.60) | 27.04 (4.51) |
| Total cholesterol (mean [SD]), mg/dL | 221.33 (43.31) | 220.00 (43.65) |
| HDL cholesterol (mean [SD]), mg/dL | 58.05 (14.95) | 57.80 (14.97) |
| HbA1c (mean [SD]), % | 5.39 (0.51) | 5.42 (0.61) |
| Systolic pressure (mean [SD]), mmHg | 136.41 (18.21) | 136.10 (18.11) |
| Diastolic pressure (mean [SD]), mmHg | 81.65 (9.93) | 81.34 (9.83) |
| Diabetes |  |  |
| No | 8263 (95.5) | 2029 (94.5) |
| Yes | 388 (4.5) | 118 (5.5) |
| Hypertension |  |  |
| No | 6642 (76.8) | 1656 (77.1) |
| Yes | 2009 (23.2) | 491 (22.9) |
| High cholesterol |  |  |
| No | 7618 (88.1) | 1886 (87.8) |
| Yes | 1033 (11.9) | 261 (12.2) |
| Insulin |  |  |
| No | 8587 (99.3) | 2126 (99.0) |
| Yes | 64 (0.7) | 21 (1.0) |
| Blood pressure medication |  |  |
| No | 7079 (81.8) | 1754 (81.7) |
| Yes | 1572 (18.2) | 393 (18.3) |
| Cholesterol lowering medication |  |  |
| No | 7239 (83.7) | 1778 (82.8) |
| Yes | 1412 (16.3) | 369 (17.2) |
| LE8 (mean [SD]) | 68.04 (11.00) | 67.96 (11.20) |
| LE8 sub-scores (mean [SD]) |  |  |
| Health behavior | 71.97 (13.42) | 71.63 (13.64) |
| Health factor | 64.11 (16.39) | 64.30 (16.39) |
| Diet | 43.11 (18.67) | 43.47 (18.31) |
| Physical Activity | 75.63 (35.16) | 75.85 (34.86) |
| Smoking | 78.86 (29.76) | 77.79 (30.74) |
| Sleeping | 90.28 (17.59) | 89.40 (18.52) |
| BMI | 70.62 (27.84) | 71.07 (27.44) |
| Non-HDL cholesterol | 48.48 (28.74) | 48.97 (28.71) |
| Glucose | 92.14 (18.21) | 91.11 (19.60) |
| Blood Pressure | 45.20 (32.77) | 46.04 (32.63) |

Abbreviations: SD, standard deviation; LE8, Life’s Essential 8; BMI, body-mass index; HDL, high-density lipoprotein.

**Table S4.** Frequency of Missing Data of Covariates.

| Variable | Frequency of missing |
| --- | --- |
| Age, mean (SD), years | 0.00% |
| Sex | 0.00% |
| Annual household income, pounds | 10.80% |
| Ethnicity | 0.30% |
| Education level | 0.50% |
| Smoking status | 0.00% |
| Alcohol status | 0.00% |

Abbreviations: SD, standard deviation.

**Table S5.** Characteristics of the test set at initial assessment of UK Biobank stratified by the RetiLE8 score.

| Variables | RetiLE8 score | | | |
| --- | --- | --- | --- | --- |
|  | Q1 | Q2 | Q3 | Q4 |
| Number of participants (N=25750) | 6438 | 6437 | 6437 | 6438 |
| Age (mean[SD]), years | 60.89 (6.41) | 58.74 (7.09) | 55.49 (7.62) | 50.39 (7.31) |
| Sex |  |  |  |  |
| Male | 3534 (54.9) | 3040 (47.2) | 2749 (42.7) | 2434 (37.8) |
| Female | 2904 (45.1) | 3397 (52.8) | 3688 (57.3) | 4004 (62.2) |
| Annual household income, pounds |  |  |  |  |
| <18,000 | 1336 (23.6) | 1118 (19.7) | 851 (14.8) | 632 (10.7) |
| 18,000-30,999 | 1636 (28.9) | 1437 (25.4) | 1317 (22.9) | 1057 (17.9) |
| 31,000-51,999 | 1471 (26.0) | 1501 (26.5) | 1571 (27.3) | 1616 (27.4) |
| 52,000-100,000 | 959 (17.0) | 1211 (21.4) | 1510 (26.3) | 1841 (31.2) |
| >100,000 | 255 (4.5) | 401 (7.1) | 502 (8.7) | 759 (12.9) |
| Ethnicity |  |  |  |  |
| White | 5817 (90.6) | 5976 (93.1) | 6060 (94.4) | 6132 (95.5) |
| Black | 246 (3.8) | 148 (2.3) | 79 (1.2) | 37 (0.6) |
| South Asian | 199 (3.1) | 163 (2.5) | 138 (2.1) | 111 (1.7) |
| Other | 157 (2.4) | 133 (2.1) | 142 (2.2) | 144 (2.2) |
| Education level |  |  |  |  |
| Low | 2226 (34.8) | 1935 (30.2) | 1615 (25.2) | 1244 (19.4) |
| Intermediate | 2080 (32.5) | 2099 (32.8) | 2150 (33.6) | 2244 (35.0) |
| High | 2089 (32.7) | 2363 (36.9) | 2637 (41.2) | 2930 (45.7) |
| Alcohol status |  |  |  |  |
| Never | 298 (4.6) | 261 (4.1) | 231 (3.6) | 233 (3.6) |
| Previous | 194 (3.0) | 187 (2.9) | 184 (2.9) | 221 (3.4) |
| Current | 5943 (92.4) | 5986 (93.0) | 6021 (93.6) | 5982 (92.9) |
| BMI (mean [SD]), kg/m² | 28.13 (4.58) | 27.52 (4.59) | 27.05 (4.62) | 25.97 (4.31) |
| Total cholesterol (mean [SD]), mg/dL | 219.36 (45.12) | 222.55 (43.61) | 222.31 (43.33) | 217.52 (40.63) |
| HDL cholesterol (mean [SD]), mg/dL | 56.48 (15.18) | 58.02 (15.31) | 58.40 (15.17) | 59.09 (14.61) |
| HbA1c (mean [SD]), % | 5.52 (0.63) | 5.45 (0.53) | 5.38 (0.52) | 5.26 (0.42) |
| Systolic pressure (mean [SD]), mmHg | 144.27 (18.14) | 140.47 (17.86) | 134.93 (16.67) | 126.95 (15.40) |
| Diastolic pressure (mean [SD]), mmHg | 84.44 (10.09) | 83.14 (10.03) | 81.31 (9.51) | 77.89 (9.13) |
| Diabetes |  |  |  |  |
| No | 5956 (92.5) | 6118 (95.0) | 6187 (96.1) | 6305 (97.9) |
| Yes | 482 (7.5) | 319 (5.0) | 250 (3.9) | 133 (2.1) |
| Hypertension |  |  |  |  |
| No | 3959 (61.5) | 4544 (70.6) | 5092 (79.1) | 5750 (89.3) |
| Yes | 2479 (38.5) | 1893 (29.4) | 1345 (20.9) | 688 (10.7) |
| High cholesterol |  |  |  |  |
| No | 5248 (81.5) | 5498 (85.4) | 5755 (89.4) | 6111 (94.9) |
| Yes | 1190 (18.5) | 939 (14.6) | 682 (10.6) | 327 (5.1) |
| Insulin |  |  |  |  |
| No | 6353 (98.7) | 6383 (99.2) | 6391 (99.3) | 6414 (99.6) |
| Yes | 85 (1.3) | 54 (0.8) | 46 (0.7) | 24 (0.4) |
| Blood pressure medication |  |  |  |  |
| No | 4404 (68.4) | 4981 (77.4) | 5440 (84.5) | 5968 (92.7) |
| Yes | 2034 (31.6) | 1456 (22.6) | 997 (15.5) | 470 (7.3) |
| Cholesterol lowering medication |  |  |  |  |
| No | 4685 (72.8) | 5121 (79.6) | 5522 (85.8) | 6014 (93.4) |
| Yes | 1753 (27.2) | 1316 (20.4) | 915 (14.2) | 424 (6.6) |
| LE8 (mean [SD]) | 64.42 (10.57) | 66.41 (10.66) | 68.46 (10.81) | 72.42 (10.89) |
| LE8 subscares (mean [SD]) |  |  |  |  |
| Health behavior | 71.56 (13.88) | 71.98 (13.54) | 72.01 (13.46) | 72.31 (13.38) |
| Health factor | 57.29 (14.74) | 60.83 (15.32) | 64.91 (15.85) | 72.53 (16.06) |
| Diet | 44.32 (19.43) | 43.62 (18.67) | 43.36 (18.55) | 42.07 (18.13) |
| Physical Activity | 76.06 (34.94) | 76.25 (34.81) | 76.39 (34.50) | 76.89 (33.91) |
| Smoking | 77.01 (29.63) | 78.41 (29.63) | 78.44 (30.40) | 78.68 (31.62) |
| Sleeping | 88.84 (18.96) | 89.66 (18.12) | 89.87 (17.47) | 91.60 (16.26) |
| BMI | 64.42 (28.19) | 68.39 (28.20) | 71.03 (27.88) | 77.14 (26.02) |
| Non-HDL cholesterol | 46.34 (27.97) | 46.58 (28.08) | 48.41 (28.67) | 54.07 (29.97) |
| Glucose | 87.98 (21.85) | 90.78 (19.34) | 92.94 (17.47) | 95.88 (13.53) |
| Blood Pressure | 30.43 (28.23) | 37.57 (30.20) | 47.24 (31.62) | 63.02 (31.12) |

Abbreviations: SD, standard deviation; LE8, Life’s Essential 8; BMI, body-mass index; HDL, high-density lipoprotein.

**Table S6.** Characteristics of the test set at initial assessment of UK Biobank stratified by medication use.

| Variables | Insulin | | Blood pressure medication | | Cholesterol lowering medication | |
| --- | --- | --- | --- | --- | --- | --- |
|  | No | Yes | No | Yes | No | Yes |
| Number of participants (N=25750) | 25541 | 209 | 20793 | 4957 | 21342 | 4408 |
| Age (mean [SD]), years | 56.37 (8.14) | 58.16 (8.02) | 55.33 (8.17) | 60.79 (6.40) | 55.32 (8.13) | 61.50 (6.01) |
| Sex |  |  |  |  |  |  |
| Male | 11632 (45.5) | 125 (59.8) | 8994 (43.3) | 2763 (55.7) | 9040 (42.4) | 2717 (61.6) |
| Female | 13909 (54.5) | 84 (40.2) | 11799 (56.7) | 2194 (44.3) | 12302 (57.6) | 1691 (38.4) |
| Annual household income, pounds |  |  |  |  |  |  |
| <18,000 | 3881 (17.0) | 56 (31.5) | 2861 (15.4) | 1076 (24.7) | 2937 (15.4) | 1000 (25.8) |
| 18,000-30,999 | 5405 (23.7) | 42 (23.6) | 4216 (22.6) | 1231 (28.2) | 4365 (22.8) | 1082 (28.0) |
| 31,000-51,999 | 6114 (26.8) | 45 (25.3) | 5077 (27.3) | 1082 (24.8) | 5227 (27.4) | 932 (24.1) |
| 52,000-100,000 | 5491 (24.1) | 30 (16.9) | 4758 (25.5) | 763 (17.5) | 4876 (25.5) | 645 (16.7) |
| >100,000 | 1912 (8.4) | 5 (2.8) | 1711 (9.2) | 206 (4.7) | 1705 (8.9) | 212 (5.5) |
| Ethnicity |  |  |  |  |  |  |
| White | 23806 (93.5) | 179 (86.1) | 19414 (93.6) | 4571 (92.5) | 19895 (93.4) | 4090 (93.2) |
| Black | 503 (2.0) | 7 (3.4) | 366 (1.8) | 144 (2.9) | 437 (2.1) | 73 (1.7) |
| South Asian | 596 (2.3) | 15 (7.2) | 481 (2.3) | 130 (2.6) | 463 (2.2) | 148 (3.4) |
| Other | 569 (2.2) | 7 (3.4) | 479 (2.3) | 97 (2.0) | 497 (2.3) | 79 (1.8) |
| Education level |  |  |  |  |  |  |
| Low | 6952 (27.4) | 68 (32.9) | 5248 (25.4) | 1772 (36.0) | 5404 (25.4) | 1616 (36.9) |
| Intermediate | 8500 (33.5) | 73 (35.3) | 6939 (33.5) | 1634 (33.2) | 7176 (33.8) | 1397 (31.9) |
| High | 9953 (39.2) | 66 (31.9) | 8508 (41.1) | 1511 (30.7) | 8658 (40.8) | 1361 (31.1) |
| Alcohol status |  |  |  |  |  |  |
| Never | 1002 (3.9) | 21 (10.0) | 781 (3.8) | 242 (4.9) | 812 (3.8) | 211 (4.8) |
| Previous | 777 (3.0) | 9 (4.3) | 603 (2.9) | 183 (3.7) | 624 (2.9) | 162 (3.7) |
| Current | 23753 (93.0) | 179 (85.6) | 19402 (93.3) | 4530 (91.4) | 19898 (93.3) | 4034 (91.5) |
| BMI (mean [SD]), kg/m² | 27.14 (4.57) | 30.17 (6.41) | 26.62 (4.30) | 29.45 (5.07) | 26.80 (4.49) | 28.94 (4.68) |
| Total cholesterol (mean [SD]), mg/dL | 220.83 (43.07) | 172.03 (37.08) | 224.91 (41.75) | 201.65 (44.39) | 227.85 (40.54) | 184.52 (37.59) |
| HDL cholesterol (mean [SD]), mg/dL | 58.04 (15.08) | 52.14 (16.75) | 59.05 (15.05) | 53.60 (14.51) | 59.11 (15.07) | 52.63 (14.05) |
| HbA1c (mean [SD]), % | 5.38 (0.48) | 7.72 (1.39) | 5.34 (0.44) | 5.66 (0.80) | 5.33 (0.42) | 5.77 (0.83) |
| Systolic pressure (mean [SD]), mmHg | 136.63 (18.26) | 139.60 (17.22) | 134.80 (17.96) | 144.45 (17.39) | 135.64 (18.24) | 141.57 (17.49) |
| Diastolic pressure (mean [SD]), mmHg | 81.72 (9.99) | 78.83 (11.17) | 81.06 (9.95) | 84.35 (9.80) | 81.59 (10.09) | 82.22 (9.58) |
| Diabetes |  |  |  |  |  |  |
| No | 24562 (96.2) | 4 (1.9) | 20320 (97.7) | 4246 (85.7) | 21022 (98.5) | 3544 (80.4) |
| Yes | 979 (3.8) | 205 (98.1) | 473 (2.3) | 711 (14.3) | 320 (1.5) | 864 (19.6) |
| Hypertension |  |  |  |  |  |  |
| No | 19256 (75.4) | 89 (42.6) | 18960 (91.2) | 385 (7.8) | 17446 (81.7) | 1899 (43.1) |
| Yes | 6285 (24.6) | 120 (57.4) | 1833 (8.8) | 4572 (92.2) | 3896 (18.3) | 2509 (56.9) |
| High cholesterol |  |  |  |  |  |  |
| No | 22468 (88.0) | 144 (68.9) | 19215 (92.4) | 3397 (68.5) | 21035 (98.6) | 1577 (35.8) |
| Yes | 3073 (12.0) | 65 (31.1) | 1578 (7.6) | 1560 (31.5) | 307 (1.4) | 2831 (64.2) |
| RetiLE8 (mean [SD]) | 68.42 (3.28) | 67.07 (2.94) | 68.77 (3.34) | 66.88 (2.51) | 68.71 (3.34) | 66.95 (2.52) |
| LE8 (mean [SD]) | 68.01 (11.08) | 57.52 (12.80) | 69.38 (10.80) | 61.82 (10.40) | 68.86 (10.94) | 63.40 (10.93) |
| LE8 sub-scores (mean [SD]) |  |  |  |  |  |  |
| Health behavior | 71.99 (13.54) | 69.10 (16.49) | 72.25 (13.38) | 70.79 (14.28) | 72.25 (13.39) | 70.59 (14.32) |
| Health factor | 64.04 (16.43) | 45.94 (16.11) | 66.52 (15.97) | 52.86 (13.96) | 65.47 (16.33) | 56.22 (15.12) |
| Diet | 43.30 (18.70) | 48.83 (19.88) | 43.09 (18.68) | 44.39 (18.83) | 43.11 (18.64) | 44.49 (19.04) |
| Physical Activity | 76.48 (34.47) | 65.55 (41.67) | 77.11 (33.98) | 73.39 (36.65) | 77.16 (33.93) | 72.72 (37.13) |
| Smoking | 78.14 (30.35) | 77.78 (29.36) | 78.43 (30.60) | 76.89 (29.17) | 78.44 (30.64) | 76.64 (28.79) |
| Sleeping | 90.04 (17.70) | 84.26 (23.40) | 90.35 (17.40) | 88.47 (19.12) | 90.29 (17.47) | 88.52 (19.03) |
| BMI | 70.37 (27.89) | 54.90 (33.11) | 73.38 (26.65) | 57.11 (29.52) | 72.41 (27.39) | 59.74 (28.38) |
| Non-HDL cholesterol | 48.69 (28.83) | 68.23 (24.99) | 47.83 (29.08) | 53.14 (27.48) | 47.37 (29.06) | 56.04 (26.67) |
| Glucose | 92.42 (17.63) | 28.28 (15.16) | 94.00 (15.63) | 83.07 (25.73) | 94.56 (14.69) | 78.98 (27.55) |
| Blood Pressure | 44.66 (32.68) | 32.34 (30.51) | 50.87 (31.62) | 18.13 (22.13) | 47.55 (32.65) | 30.11 (28.75) |

Abbreviations: SD, standard deviation; LE8, Life’s Essential 8; BMI, body-mass index; HDL, high-density lipoprotein.

**Table S7.** Associations of Life’s Essential 8 with all-cause mortality, cardiovascular mortality and cardiovascular disease events.

| Outcomes | LE8 | n/N | LE8 Mean(Range) | RetiLE8 Mean(Range) | Crude model ^a^ | | Adjusted model ^b^ | |
| --- | --- | --- | --- | --- | --- | --- | --- | --- |
|  |  |  |  |  | HR(95% CI) | *P* | HR(95% CI) | *P* |
| All-cause mortality | Q1 | 538/6750 | 53.73(18.75-60.62) | 67.44(59.48-80.39) | 1.00(ref) |  | 1.00(ref) |  |
|  | Q2 | 374/6236 | 64.91(61.25-68.12) | 67.85(60.16-81.29) | 0.74(0.65,0.85) | <0.001 | 0.78(0.68,0.89) | <0.001 |
|  | Q3 | 294/6447 | 72.08(68.75-75.62) | 68.56(60.36-83.14) | 0.56(0.49,0.65) | <0.001 | 0.64(0.56,0.74) | <0.001 |
|  | Q4 | 219/6317 | 81.84(76.25-100.00) | 69.84(60.29-84.41) | 0.42(0.36,0.50) | <0.001 | 0.66(0.56,0.78) | <0.001 |
|  | Per 1-SD increase ^c^ | | | | 0.71(0.67,0.75) | <0.001 | 0.80(0.76,0.85) | <0.001 |
| Cardiovascular disease mortality | Q1 | 97/6750 | 53.73(18.75-60.62) | 67.44(59.48-80.39) | 1.00(ref) |  | 1.00(ref) |  |
|  | Q2 | 57/6236 | 64.91(61.25-68.12) | 67.85(60.16-81.29) | 0.63(0.45,0.87) | 0.005 | 0.66(0.48,0.92) | 0.01 |
|  | Q3 | 38/6447 | 72.08(68.75-75.62) | 68.56(60.36-83.14) | 0.40(0.28,0.58) | <0.001 | 0.48(0.33,0.70) | <0.001 |
|  | Q4 | 21/6317 | 81.84(76.25-100.00) | 69.84(60.29-84.41) | 0.23(0.14,0.36) | <0.001 | 0.39(0.25,0.64) | <0.001 |
|  | Per 1-SD increase ^c^ | | | | 0.57(0.51,0.65) | <0.001 | 0.65(0.57,0.75) | <0.001 |
| Cardiovascular disease events | Q1 | 892/6324 | 53.83(18.75-60.62) | 67.48(59.48-80.39) | 1.00(ref) |  | 1.00(ref) |  |
|  | Q2 | 641/5966 | 64.91(61.25-68.12) | 67.89(60.16-81.29) | 0.74(0.67,0.82) | <0.001 | 0.77(0.69,0.85) | <0.001 |
|  | Q3 | 561/6174 | 72.07(68.75-75.62) | 68.61(60.36-83.14) | 0.62(0.55,0.68) | <0.001 | 0.70(0.63,0.78) | <0.001 |
|  | Q4 | 334/6148 | 81.87(76.25-100.00) | 69.88(60.29-84.41) | 0.36(0.32,0.41) | <0.001 | 0.53(0.47,0.61) | <0.001 |
|  | Per 1-SD increase ^c^ | | | | 0.69(0.67,0.72) | <0.001 | 0.77(0.74,0.80) | <0.001 |

Abbreviations: LE8, Life’s Essential 8; SD, SD, standard deviation; HR, hazard ratio; Q1-Q4, quartile 1- quartile 4.

^a^ Crude models.

^b^ Adjusted for age, sex, annual household income, ethnicity, education level, alcohol status.

^c^ Life’s Essential 8 scores were Z-score transformed to assess the association per 1-SD increase with the outcome.

**Table S8.** Joint associations of Life’s Essential 8 and RetiLE8 with all-cause mortality, cardiovascular mortality and cardiovascular disease events.

| Outcomes | Category ^a^ | n/N | LE8 Mean(Range) | RetiLE8 Mean(Range) | Crude model ^b^ | | Adjusted model ^c^ | |
| --- | --- | --- | --- | --- | --- | --- | --- | --- |
|  |  |  |  |  | HR(95% CI) | *P* | HR(95% CI) | *P* |
| All-cause mortality | Low LE8 & Low RetiLE8 | 644/7417 | 58.24(18.75-67.50) | 65.71(59.48-67.91) | 1.00(ref) |  | 1.00(ref) |  |
|  | Low LE8 & High RetiLE8 | 234/4957 | 59.27(24.38-67.50) | 70.45(67.91-81.29) | 0.53(0.46,0.62) | <0.001 | 0.92(0.79,1.08) | 0.31 |
|  | High LE8 & Low RetiLE8 | 356/5458 | 75.17(68.12-97.50) | 65.98(60.29-67.91) | 0.74(0.65,0.84) | <0.001 | 0.80(0.70,0.91) | <0.001 |
|  | High LE8 & High RetiLE8 | 191/7918 | 77.43(68.12-100.00) | 71.33(67.91-84.41) | 0.27(0.23,0.32) | <0.001 | 0.57(0.48,0.68) | <0.001 |
| Cardiovascular disease mortality | Low LE8 & Low RetiLE8 | 114/7417 | 58.24(18.75-67.50) | 65.71(59.48-67.91) | 1.00(ref) |  | 1.00(ref) |  |
|  | Low LE8 & High RetiLE8 | 32/4957 | 59.27(24.38-67.50) | 70.45(67.91-81.29) | 0.41(0.28,0.61) | <0.001 | 0.84(0.56,1.26) | 0.41 |
|  | High LE8 & Low RetiLE8 | 50/5458 | 75.17(68.12-97.50) | 65.98(60.29-67.91) | 0.59(0.42,0.82) | 0.002 | 0.66(0.47,0.93) | 0.02 |
|  | High LE8 & High RetiLE8 | 17/7918 | 77.43(68.12-100.00) | 71.33(67.91-84.41) | 0.13(0.08,0.22) | <0.001 | 0.36(0.21,0.60) | <0.001 |
| Cardiovascular disease events | Low LE8 & Low RetiLE8 | 1059/6960 | 58.34(18.75-67.50) | 65.73(59.48-67.91) | 1.00(ref) |  | 1.00(ref) |  |
|  | Low LE8 & High RetiLE8 | 424/4745 | 59.38(24.38-67.50) | 70.48(67.91-81.29) | 0.56(0.50,0.63) | <0.001 | 0.89(0.79,1.00) | 0.05 |
|  | High LE8 & Low RetiLE8 | 543/5177 | 75.21(68.12-97.50) | 65.99(60.29-67.91) | 0.66(0.60,0.74) | <0.001 | 0.73(0.65,0.81) | <0.001 |
|  | High LE8 & High RetiLE8 | 402/7730 | 77.47(68.12-100.00) | 71.35(67.91-84.41) | 0.32(0.28,0.35) | <0.001 | 0.60(0.53,0.68) | <0.001 |

Abbreviations: LE8, Life’s Essential 8; HR, hazard ratio.

^a^ The LE8 and RetiLE8 scores were dichotomized into low and high groups based on their respective medians.

^b^ Crude models.

^c^ Adjusted for age, sex, annual household income, ethnicity, education level, alcohol status.

**Table S9.** Comparison of discrimination and reclassification among pooled cohort equations, RetiLE8, and their combination.

| Outcomes | Model | Concordance(95% CI) | ΔC index(95% CI) | *P* | NRI(95% CI) | *P* |
| --- | --- | --- | --- | --- | --- | --- |
| All-cause mortality | PCE | 0.727(0.715,0.740) | 0(ref) |  | 0(ref) |  |
|  | RetiLE8 | 0.727(0.715,0.740) | 0.000(-0.002,0.003) | 0.91 | 6.4%(0.9%,10.1%) | 0.02 |
|  | PCE+RetiLE8 | 0.730(0.717,0.743) | 0.003(0.001,0.004) | 0.004 | 5.6%(0.7%,10.5%) | 0.02 |
| Cardiovascular disease mortality | PCE | 0.814(0.788,0.840) | 0(ref) |  | 0(ref) |  |
|  | RetiLE8 | 0.810(0.783,0.836) | -0.004(-0.010,0.002) | 0.19 | 7.6%(-5.9%,20.0%) | 0.28 |
|  | PCE+RetiLE8 | 0.816(0.791,0.842) | 0.002(-0.001,0.006) | 0.18 | 7.8%(-4.4%,21.9%) | 0.20 |
| Cardiovascular disease events | PCE | 0.706(0.696,0.716) | 0(ref) |  | 0(ref) |  |
|  | RetiLE8 | 0.703(0.693,0.713) | -0.003(-0.005,-0.001) | 0.008 | 5.2%(2.1%,8.9%) | 0.006 |
|  | PCE+RetiLE8 | 0.708(0.698,0.718) | 0.002(0.000,0.004) | 0.01 | 4.4%(0.6%,8.1%) | 0.03 |

All models included age, sex, annual household income, ethnicity, education level, alcohol status. PCE, pooled cohort equations; ΔC index, difference in the Harrell’s concordance index; NRI, net reclassification improvement index.

**Table S10.** Comparison of discrimination and reclassification among the Life’s Essential 8, RetiLE8, and their combination excluding participants receiving cholesterol lowering medication.

| Outcomes | Model | Concordance(95% CI) | ΔC index(95% CI) | *P* | NRI(95% CI) | *P* |
| --- | --- | --- | --- | --- | --- | --- |
| All-cause mortality | LE8 | 0.722(0.706,0.738) | 0(ref) |  | 0(ref) |  |
|  | RetiLE8 | 0.721(0.706,0.737) | -0.001(-0.005,0.003) | 0.68 | -0.1%(-6.3%,6.1%) | 0.94 |
|  | LE8+RetiLE8 | 0.725(0.709,0.740) | 0.003(0.000,0.005) | 0.02 | 5.9%(0.0%,11.2%) | 0.05 |
| Cardiovascular disease mortality | LE8 | 0.811(0.773,0.848) | 0(ref) |  | 0(ref) |  |
|  | RetiLE8 | 0.806(0.767,0.845) | -0.005(-0.015,0.006) | 0.38 | 1.3%(-20.3%,18.8%) | 0.90 |
|  | LE8+RetiLE8 | 0.814(0.777,0.851) | 0.003(-0.002,0.009) | 0.26 | 9.0%(-7.8%,23.1%) | 0.28 |
| Cardiovascular disease events | LE8 | 0.710(0.698,0.721) | 0(ref) |  | 0(ref) |  |
|  | RetiLE8 | 0.705(0.693,0.717) | -0.005(-0.008,-0.001) | 0.01 | -4.6%(-9.2%,0.2%) | 0.06 |
|  | LE8+RetiLE8 | 0.711(0.700,0.723) | 0.002(0.000,0.003) | 0.04 | 5.6%(1.0%,9.3%) | 0.006 |

All models included age, sex, annual household income, ethnicity, education level, alcohol status. LE8, Life’s Essential 8; ΔC index, difference in the Harrell’s concordance index; NRI, net reclassification improvement index.

**Table S11.** Comparison of discrimination and reclassification among pooled cohort equations, RetiLE8, and their combination excluding participants receiving cholesterol lowering medication.

| Outcomes | Model | Concordance(95% CI) | ΔC index(95% CI) | *P* | NRI(95% CI) | *P* |
| --- | --- | --- | --- | --- | --- | --- |
| All-cause mortality | PCE | 0.720(0.704,0.736) | 0(ref) |  | 0(ref) |  |
|  | RetiLE8 | 0.721(0.706,0.737) | 0.001(-0.002,0.004) | 0.55 | 7.2%(1.7%,11.4%) | 0.008 |
|  | PCE+RetiLE8 | 0.724(0.708,0.739) | 0.004(0.001,0.006) | 0.007 | 5.6%(0.2%,11.7%) | 0.04 |
| Cardiovascular disease mortality | PCE | 0.810(0.771,0.849) | 0(ref) |  | 0(ref) |  |
|  | RetiLE8 | 0.806(0.767,0.845) | -0.004(-0.014,0.006) | 0.40 | 8.1%(-9.7%,26.0%) | 0.37 |
|  | PCE+RetiLE8 | 0.815(0.777,0.852) | 0.004(-0.003,0.011) | 0.26 | 10.6%(-5.7%,27.3%) | 0.25 |
| Cardiovascular disease events | PCE | 0.708(0.697,0.720) | 0(ref) |  | 0(ref) |  |
|  | RetiLE8 | 0.705(0.693,0.717) | -0.003(-0.006,-0.001) | 0.006 | 5.3%(1.0%,9.5%) | 0.010 |
|  | PCE+RetiLE8 | 0.710(0.699,0.722) | 0.002(-0.000,0.004) | 0.06 | 5.5%(0.8%,9.8%) | 0.010 |

All models included age, sex, annual household income, ethnicity, education level, alcohol status. PCE, pooled cohort equations; ΔC index, difference in the Harrell’s concordance index; NRI, net reclassification improvement index.

**Table S12.** Pearson’s correlations of LE8 and RetiLE8 scores with age, individual LE8 components, and related variables.

| Variables | LE8 | | RetiLE8 | |
| --- | --- | --- | --- | --- |
|  | r (95% CI) | *P* | r (95% CI) | *P* |
| Age | -0.18(-0.19,-0.17) | <0.001 | -0.51(-0.51,-0.50) | <0.001 |
| BMI | -0.57(-0.58,-0.57) | <0.001 | -0.18(-0.20,-0.17) | <0.001 |
| Total cholesterol | -0.23(-0.24,-0.22) | <0.001 | -0.04(-0.05,-0.02) | <0.001 |
| HDL cholesterol | 0.25(0.24,0.26) | <0.001 | 0.06(0.05,0.07) | <0.001 |
| HbA1c | -0.32(-0.33,-0.31) | <0.001 | -0.18(-0.20,-0.17) | <0.001 |
| Systolic pressure | -0.44(-0.45,-0.43) | <0.001 | -0.38(-0.39,-0.37) | <0.001 |
| Diastolic pressure | -0.45(-0.46,-0.44) | <0.001 | -0.27(-0.28,-0.26) | <0.001 |
| LE8 subscares |  |  |  |  |
| Health behavior | 0.67(0.67,0.68) | <0.001 | 0.02(0.01,0.03) | 0.002 |
| Health factor | 0.79(0.79,0.80) | <0.001 | 0.37(0.36,0.38) | <0.001 |
| Diet | 0.27(0.26,0.28) | <0.001 | -0.04(-0.05,-0.03) | <0.001 |
| Physical Activity | 0.47(0.46,0.48) | <0.001 | 0.01(-0.00,0.02) | 0.08 |
| Smoking | 0.35(0.34,0.36) | <0.001 | 0.01(0.00,0.03) | 0.03 |
| Sleeping | 0.26(0.25,0.27) | <0.001 | 0.06(0.05,0.07) | <0.001 |
| BMI | 0.58(0.57,0.59) | <0.001 | 0.18(0.16,0.19) | <0.001 |
| Non-HDL cholesterol | 0.43(0.42,0.44) | <0.001 | 0.12(0.11,0.13) | <0.001 |
| Glucose | 0.35(0.34,0.36) | <0.001 | 0.16(0.15,0.17) | <0.001 |
| Blood Pressure | 0.53(0.52,0.54) | <0.001 | 0.40(0.39,0.41) | <0.001 |

Abbreviations: LE8, Life’s Essential 8; r, Pearson’s correlation coefficient; BMI, body-mass index; HDL, high-density lipoprotein.

**Table S13.** Pearson’s correlations of LE8 scores with age, individual LE8 components, and related variables stratified by LE8 tertiles.

| Variables | T1 | | T2 | | T3 | |
| --- | --- | --- | --- | --- | --- | --- |
|  | r (95% CI) | *P* | r (95% CI) | *P* | r (95% CI) | *P* |
| Age | 0.01(-0.01,0.03) | 0.52 | -0.03(-0.05,-0.00) | 0.02 | -0.21(-0.23,-0.19) | <0.001 |
| BMI | -0.34(-0.36,-0.32) | <0.001 | -0.16(-0.18,-0.14) | <0.001 | -0.29(-0.31,-0.27) | <0.001 |
| Total cholesterol | -0.03(-0.05,-0.01) | 0.004 | -0.07(-0.09,-0.05) | <0.001 | -0.26(-0.28,-0.24) | <0.001 |
| HDL cholesterol | 0.13(0.11,0.15) | <0.001 | 0.07(0.05,0.09) | <0.001 | 0.08(0.06,0.10) | <0.001 |
| HbA1c | -0.25(-0.27,-0.23) | <0.001 | -0.07(-0.10,-0.05) | <0.001 | -0.12(-0.15,-0.10) | <0.001 |
| Systolic pressure | -0.09(-0.11,-0.07) | <0.001 | -0.13(-0.15,-0.11) | <0.001 | -0.39(-0.41,-0.37) | <0.001 |
| Diastolic pressure | -0.13(-0.15,-0.11) | <0.001 | -0.14(-0.16,-0.12) | <0.001 | -0.36(-0.38,-0.34) | <0.001 |
| LE8 subscares |  |  |  |  |  |  |
| Health behavior | 0.55(0.53,0.56) | <0.001 | 0.20(0.18,0.22) | <0.001 | 0.33(0.31,0.34) | <0.001 |
| Health factor | 0.46(0.44,0.48) | <0.001 | 0.31(0.30,0.33) | <0.001 | 0.67(0.65,0.68) | <0.001 |
| Diet | 0.12(0.10,0.14) | <0.001 | 0.06(0.04,0.09) | <0.001 | 0.17(0.15,0.19) | <0.001 |
| Physical Activity | 0.37(0.35,0.39) | <0.001 | 0.11(0.09,0.13) | <0.001 | 0.16(0.13,0.18) | <0.001 |
| Smoking | 0.21(0.19,0.23) | <0.001 | 0.10(0.08,0.12) | <0.001 | 0.18(0.16,0.20) | <0.001 |
| Sleeping | 0.20(0.18,0.22) | <0.001 | 0.07(0.05,0.09) | <0.001 | 0.10(0.08,0.12) | <0.001 |
| BMI | 0.33(0.31,0.34) | <0.001 | 0.17(0.15,0.19) | <0.001 | 0.28(0.26,0.30) | <0.001 |
| Non-HDL cholesterol | 0.12(0.10,0.14) | <0.001 | 0.13(0.11,0.15) | <0.001 | 0.40(0.38,0.42) | <0.001 |
| Glucose | 0.26(0.24,0.28) | <0.001 | 0.08(0.06,0.10) | <0.001 | 0.10(0.08,0.12) | <0.001 |
| Blood Pressure | 0.17(0.15,0.19) | <0.001 | 0.16(0.14,0.18) | <0.001 | 0.43(0.41,0.44) | <0.001 |

Abbreviations: LE8, Life’s Essential 8; T1-T3, tertile 1 - tertile 3; r, Pearson’s correlation coefficient; BMI, body-mass index; HDL, high-density lipoprotein.

**Table S14.** Pearson’s correlations of RetiLE8 scores with age, individual LE8 components, and related variables stratified by RetiLE8 tertiles.

| Variables | T1 | | T2 | | T3 | |
| --- | --- | --- | --- | --- | --- | --- |
|  | r (95% CI) | *P* | r (95% CI) | *P* | r (95% CI) | *P* |
| Age | -0.10(-0.12,-0.08) | <0.001 | -0.17(-0.19,-0.14) | <0.001 | -0.33(-0.35,-0.31) | <0.001 |
| BMI | -0.06(-0.08,-0.04) | <0.001 | -0.03(-0.05,-0.00) | 0.02 | -0.14(-0.16,-0.12) | <0.001 |
| Total cholesterol | 0.04(0.02,0.06) | <0.001 | -0.00(-0.02,0.02) | 0.94 | -0.10(-0.12,-0.08) | <0.001 |
| HDL cholesterol | 0.04(0.02,0.06) | <0.001 | 0.01(-0.01,0.03) | 0.49 | 0.02(-0.00,0.04) | 0.11 |
| HbA1c | -0.06(-0.08,-0.04) | <0.001 | -0.03(-0.05,-0.01) | 0.007 | -0.11(-0.13,-0.09) | <0.001 |
| Systolic pressure | -0.08(-0.10,-0.06) | <0.001 | -0.10(-0.12,-0.08) | <0.001 | -0.26(-0.28,-0.24) | <0.001 |
| Diastolic pressure | -0.08(-0.10,-0.05) | <0.001 | -0.06(-0.08,-0.04) | <0.001 | -0.21(-0.23,-0.19) | <0.001 |
| LE8 subscares |  |  |  |  |  |  |
| Health behavior | 0.03(0.01,0.05) | 0.002 | 0.01(-0.01,0.03) | 0.26 | 0.02(-0.00,0.04) | 0.07 |
| Health factor | 0.09(0.07,0.11) | <0.001 | 0.08(0.06,0.10) | <0.001 | 0.25(0.23,0.27) | <0.001 |
| Diet | 0.00(-0.02,0.02) | 0.81 | 0.01(-0.02,0.03) | 0.59 | -0.02(-0.04,0.00) | 0.09 |
| Physical Activity | 0.02(-0.00,0.04) | 0.05 | 0.01(-0.01,0.03) | 0.39 | 0.02(-0.00,0.04) | 0.07 |
| Smoking | 0.02(0.00,0.04) | 0.03 | 0.00(-0.02,0.03) | 0.71 | -0.00(-0.02,0.02) | 0.75 |
| Sleeping | 0.02(-0.00,0.04) | 0.06 | 0.01(-0.02,0.03) | 0.58 | 0.05(0.03,0.07) | <0.001 |
| BMI | 0.06(0.04,0.08) | <0.001 | 0.03(0.01,0.05) | 0.005 | 0.13(0.10,0.15) | <0.001 |
| Non-HDL cholesterol | -0.02(-0.04,0.01) | 0.15 | 0.02(-0.00,0.04) | 0.07 | 0.14(0.11,0.16) | <0.001 |
| Glucose | 0.06(0.04,0.09) | <0.001 | 0.03(0.01,0.05) | 0.01 | 0.07(0.05,0.09) | <0.001 |
| Blood Pressure | 0.10(0.08,0.12) | <0.001 | 0.10(0.08,0.13) | <0.001 | 0.26(0.24,0.28) | <0.001 |

Abbreviations: T1-T3, tertile 1 - tertile 3; r, Pearson’s correlation coefficient; BMI, body-mass index; HDL, high-density lipoprotein.

**
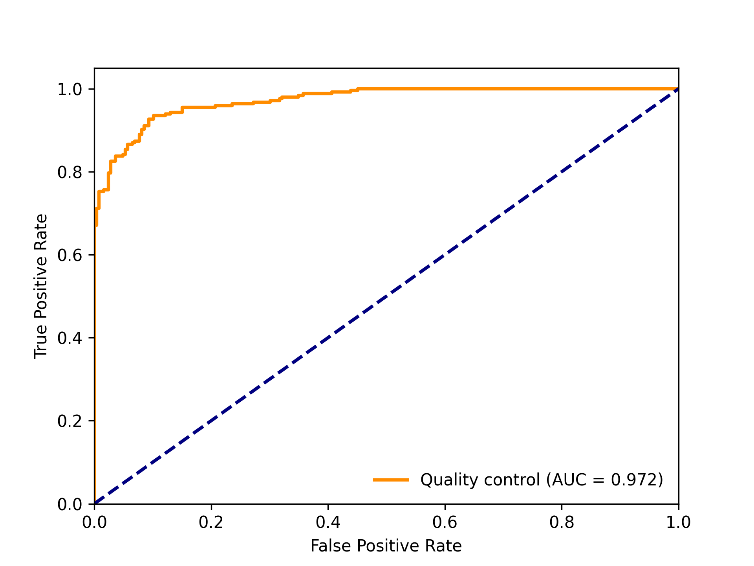
Figure S1.** Receiver operating characteristic curve of the retinal photograph quality control model.

Abbreviations: AUC, area under receiver operating characteristic curve.

**
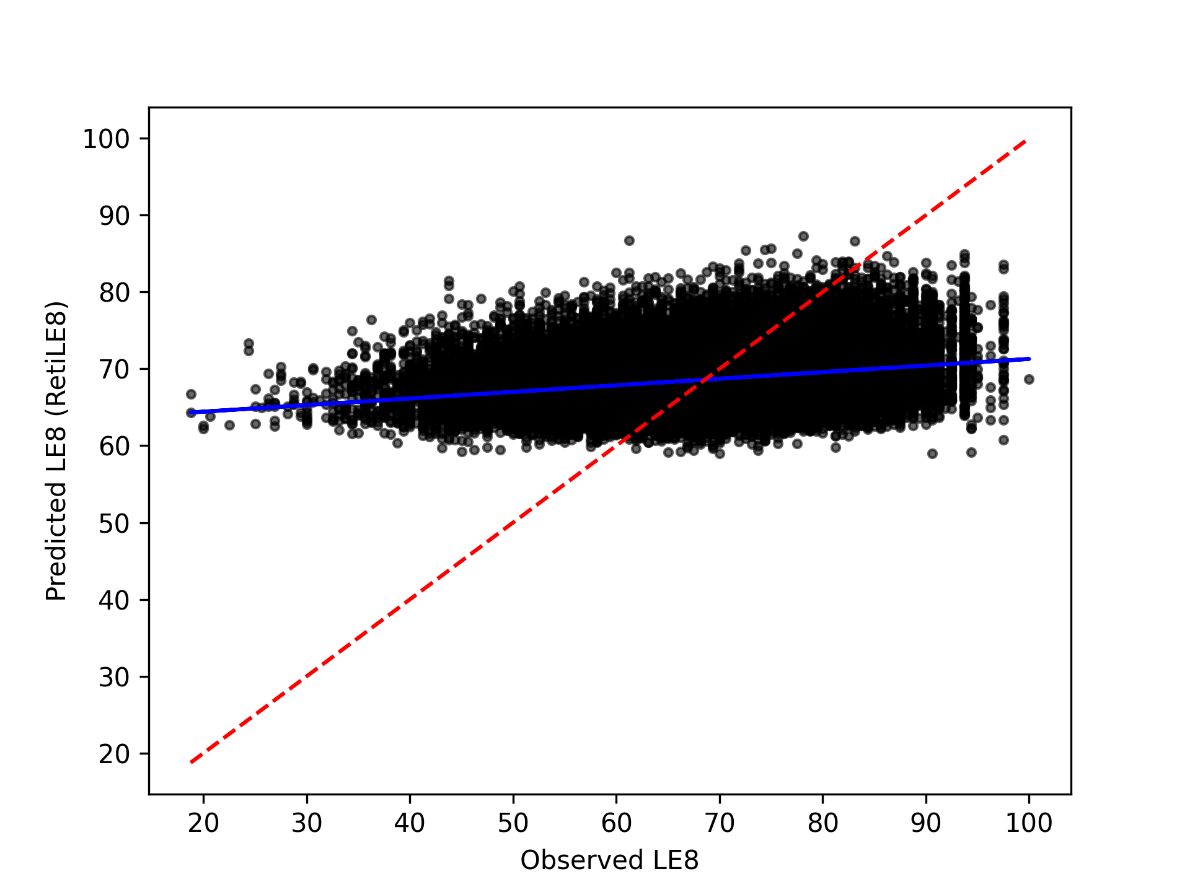
Figure S2.** Correlations between the observed Life’s Essential 8 and the predicted Retinal Life’s Essential 8 (RetiLE8).


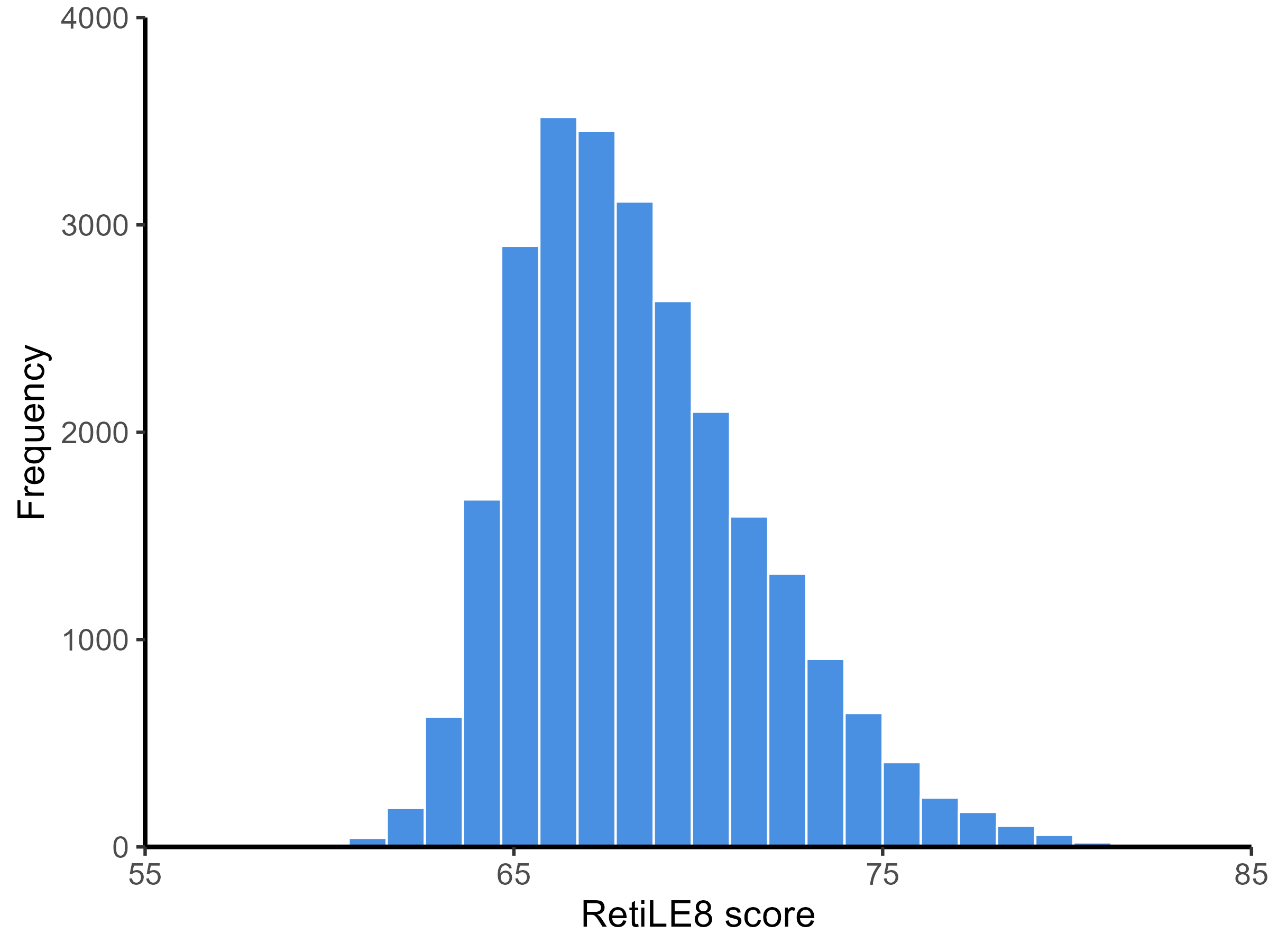
**Figure S3.** Distribution of Retinal Life’s Essential 8 (RetiLE8).

**
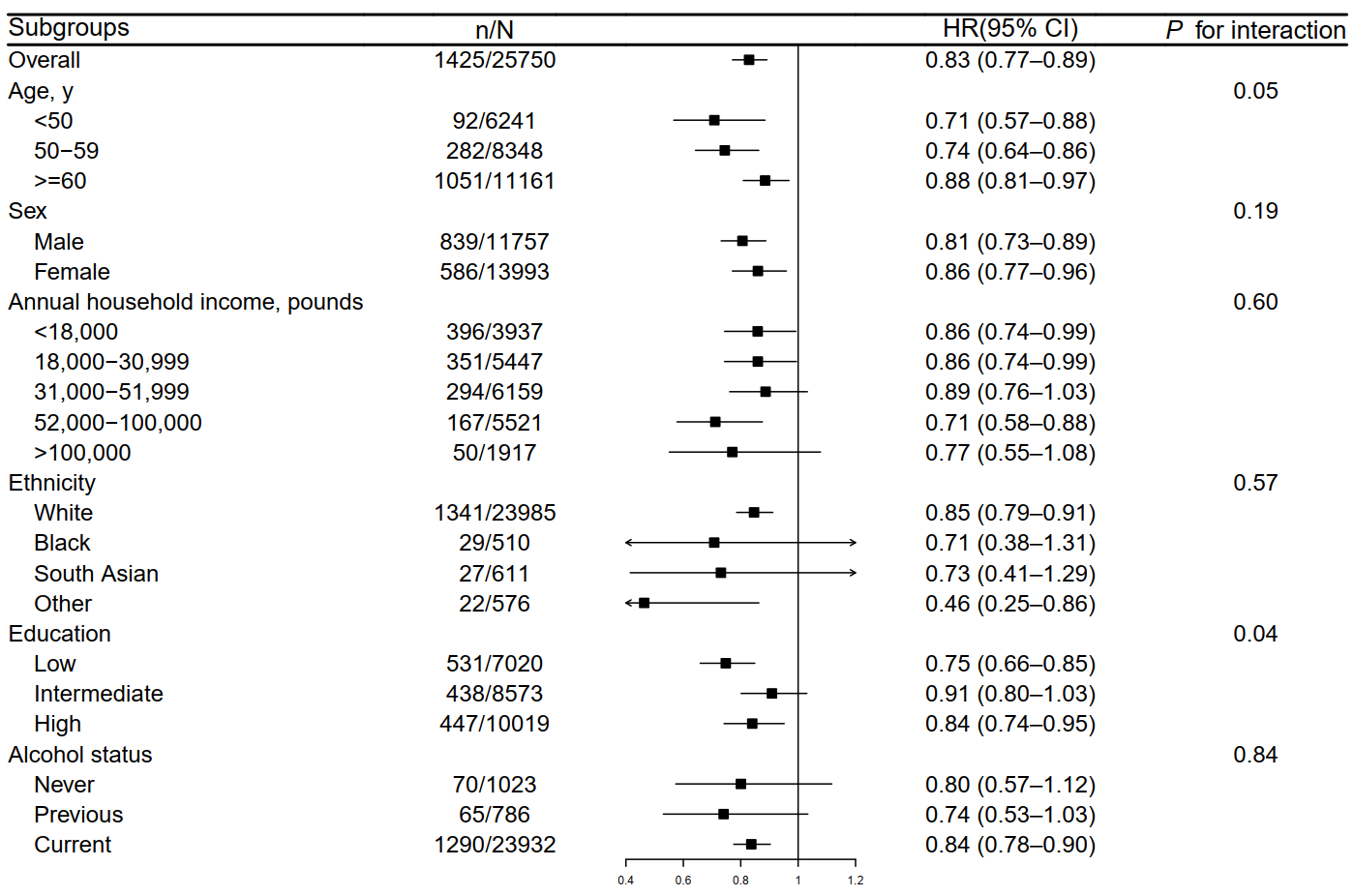
Figure S4.** Subgroup analysis for the associations between per RetiLE8 1-SD increase and all-cause mortality.

Abbreviations: HR, hazard ratio.

The models were adjusted for age, sex, annual household income, ethnicity, education level, alcohol status. Age was treated as a categorical variable when testing the interaction between age and RetiLE8, and as a continuous variable when included as a covariate.

**
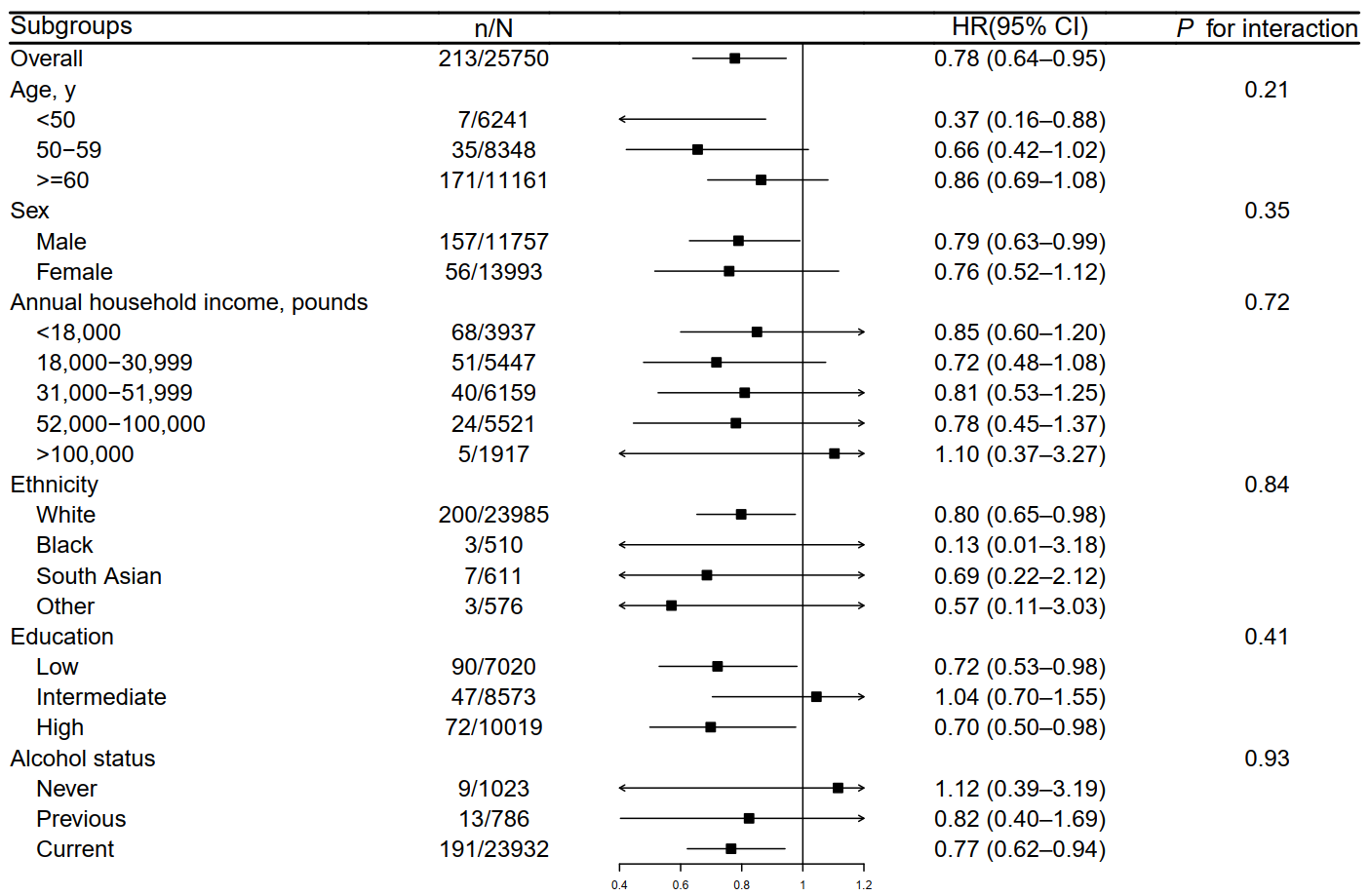
Figure S5.** Subgroup analysis for the associations between per RetiLE8 1-SD increase and cardiovascular mortality.

Abbreviations: HR, hazard ratio.

The models were adjusted for age, sex, annual household income, ethnicity, education level, alcohol status. Age was treated as a categorical variable when testing the interaction between age and RetiLE8, and as a continuous variable when included as a covariate.

**
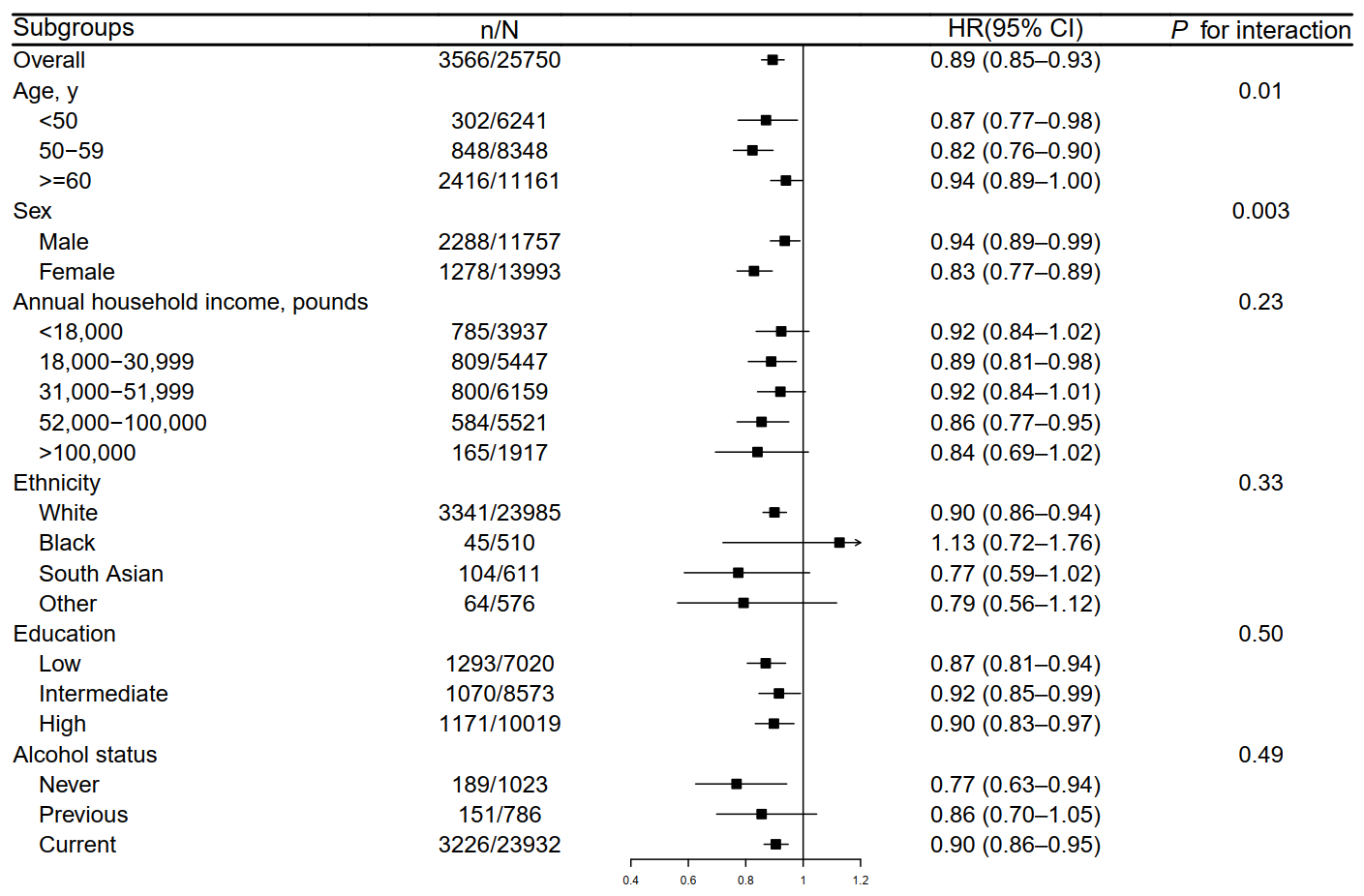
Figure S6.** Subgroup analysis for the associations between per RetiLE8 1-SD increase and cardiovascular disease events.

Abbreviations: HR, hazard ratio.

The models were adjusted for age, sex, annual household income, ethnicity, education level, alcohol status. Age was treated as a categorical variable when testing the interaction between age and RetiLE8, and as a continuous variable when included as a covariate.
